# The current landscape of television and movies in medical education

**DOI:** 10.1007/s40037-015-0205-9

**Published:** 2015-09-17

**Authors:** Marcus Law, Wilson Kwong, Farah Friesen, Paula Veinot, Stella L. Ng

**Affiliations:** 1 Department of Family & Community Medicine, Faculty of Medicine, University of Toronto, Room 2325, Medical Sciences Building, 1 King’s College Circle, M5S 1A8 Toronto, ON Canada; 2 Centre for Faculty Development, Li Ka Shing Knowledge Institute, St. Michael’s Hospital, Toronto, ON Canada; 3 Toronto East General Hospital, Toronto, ON Canada; 4 Queen’s University, Kingston, ON Canada; 5 Department of Speech-Language Pathology, University of Toronto, Toronto, Ontario Canada; 6 Centre for Ambulatory Care Education, Women’s College Hospital, Toronto, Ontario Canada

**Keywords:** Medical education, Movies, Television, Review

## Abstract

**Background:**

Using commercially available television and movies is a potentially effective tool to foster humanistic, compassionate and person-centred orientations in medical students.

**Aim:**

We reviewed pedagogical applications of television and movies in medical education to explore whether and why this innovation holds promise.

**Methods:**

We performed a literature review to provide a narrative summary on this topic.

**Results:**

Further studies are needed with richer descriptions of innovations and more rigorous research designs.

**Conclusion:**

As we move toward evidence-informed education, we need an evidence- based examination of this topic that will move it beyond a ‘show and tell’ discussion toward meaningful implementation and evaluation. Further exploration regarding the theoretical basis for using television and movies in medical education will help substantiate continued efforts to use these media as teaching tools.

**Electronic supplementary material:**

The online version of this article (doi: 10.1007/s40037-015-0205-9) contains supplementary material, which is available to authorized users.

## Essentials

- It is challenging to foster humanistic, compassionate and person-centred orientations in medical care and education.
- A potentially effective approach to this goal is the use of television and movies as educational tools.
- There is promise for television and movies to play a unique role in medical education in relation to fostering compassionate, critically conscious care orientations.
- Further exploration regarding the theoretical basis for using television and movies and objective evaluation of whether or not this intervention increases knowledge are suggested as avenues for future research.

## Introduction

Innovation in medical education continues to transform the way medical students learn in the classroom. While lecture-based teaching continues to be a staple of medical education, a variety of audio-visual media have also been introduced in the classroom setting. For example, some teachers use videos of actual or simulated events, scripted scenes for teaching purposes, and still images to enhance and support their teaching. There is evidence suggesting that, as early as the 1890s, physicians recorded patients on film for the benefit of teaching medical students [1]. The appeal of using audio-visual media is apparent, even if its effectiveness is still to be determined. Further, emotions play a specific role in learning attitudes and behaviours, and audio-visual media such as television and movies can enhance emotions, making learning more memorable and pleasurable [2, 3]. One might also speculate that a video format creates a degree of realism that is more conducive to learning about the humanistic factors in medicine [4, 5]. This is particularly true of the specific audio-visual medium of television and movies, which has been an ongoing trend in medical education over the past two decades, in addition to the influx and rising popularity of dramatized medicine in popular culture [6, 7].

A number of articles have examined the use of either television or movie clips to teach medical students about a variety of different topics. The use of television and movies in medical education may be related to, and perhaps capitalizes upon, the highly emotional context in which medical education takes place. Medical education research has shown that students are more likely to learn and remember something when the teaching point is associated with an emotional response [3, 8]. Outside of medicine, there is also a great deal of evidence that being in an emotional state can influence one’s learning and memory pattern [9].

It is challenging to foster humanistic, compassionate and person-centred orientations in medical care and education [10], and we propose that a potentially effective approach to this goal is the use of television and movies as educational tools [2, 3]. Although we hypothesize that emotions play a role in explaining why television and movies serve as an effective teaching tool, the pedagogical characteristics of this teaching format have not been formally evaluated in the academic literature. In line with efforts to ensure that innovation in medical education is evidence-informed [11], we sought to conduct a literature review on pedagogical applications of television and movies in medical education to explore why this innovation is so captivating for medical trainees. We examined the available evidence regarding why and how television and movies are used as a pedagogical modality, the various topics being taught, and the student outcomes and evaluations. Our goal was to better understand the utility of this modality and, in doing so, identify both its strength and weaknesses as a teaching tool, given that educators are likely to continue its use in medical education.

## Methods

To critically evaluate the current landscape of the use of television and movies in medical education, we performed a literature review to provide a narrative summary on this topic [12].

### Search strategy

We performed searches in PubMed and OVID Medline databases from inception to 21 October 2014, using a combination of MeSH (education, medical; students, medical; faculty, medical; motion pictures as topic; television) and keyword searches (medical education; movie*; motion picture; drama; TV; television; cinemeducation). We also reviewed the tables of content of select journals from January 2000 to October 2014, including early online publications (*Medical Teacher, Medical Education, Teaching and Learning in Medicine, Advances in Medical Education and Practice* and *Academic Medicine)*. We footnote chased the reference lists of articles included for full-text review, which were imported into a citation management system (EndNote) with any duplicates removed.

### Article selection

**Fig. 1:**
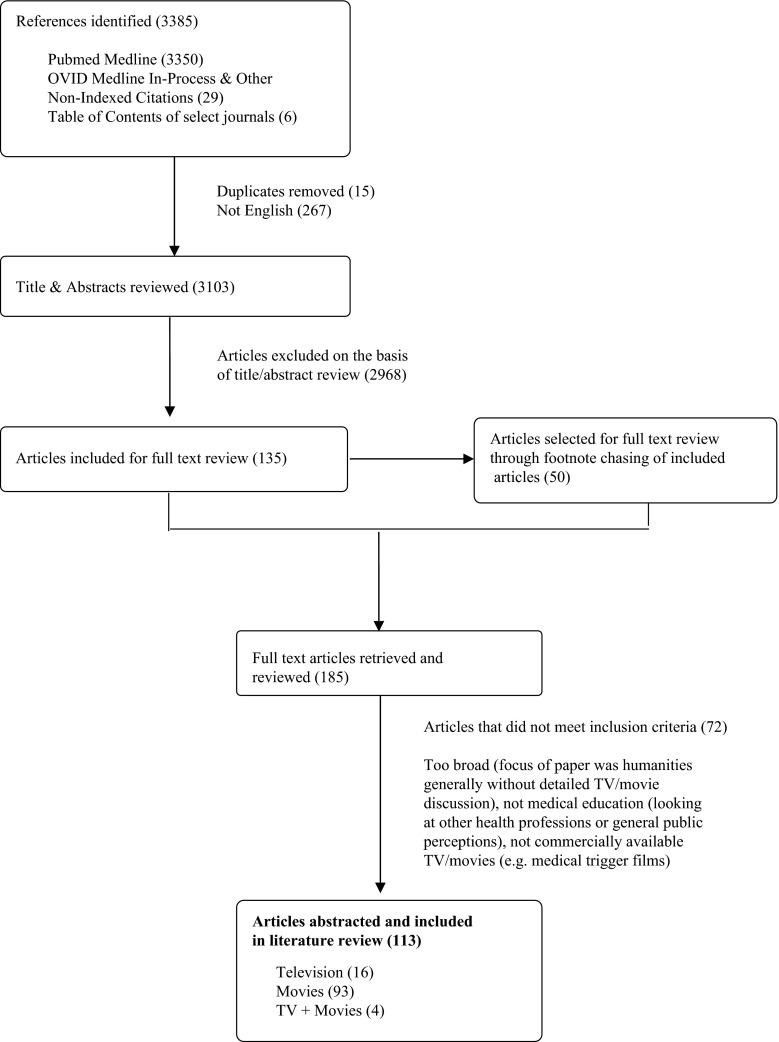
Search Strategy Flow Chart

Two authors (WK, FF) identified relevant articles for full-text review by examining the titles and abstracts of the search results. We included research, review and opinion articles in English. Articles were included if they discussed the use of *commercially* available television and movies (including documentaries) in medical education. Articles related to non-medical health professions education and the influence of television and movies on public perception were excluded. Articles discussing medical students’ television or movie viewing habits, or why these media are interesting were excluded, as the main focus of the article is how these media are used *to educate* medical students. Articles which only briefly mentioned television or movies in the context of a general discussion on humanities in health care were also excluded (Fig. 1). Each article included for full-text review was independently reviewed with data abstracted by two authors (WK, FF). Data abstraction fields included authors, title, journal, year of publication, intervention (television or movies), purpose of intervention, study design, participants, outcomes, key findings, and Kirkpatrick’s four levels of change model [13].

## Results

Of the 113 articles included in this narrative summary, 16 articles discussed television shows, 93 articles concerned movies, and 4 articles discussed both television and movies. We present our findings organized according to our questions for this review. In relation to television and movies in medical education, we used the following questions: What purpose did they serve? What content was presented? In what formats were they used? What were the outcomes or evaluation results?

### Purpose (Why?)

The papers reviewed described television and movies being used in medical education to convey factual information to attain knowledge, facilitate skills acquisition, enrich learning, maintain interest and engagement, encourage debate and discussion, motivate reflection, evoke emotion and stimulate memory retention. These purposes were similar for both media.

### Topics/Content (What?)

With respect to television, the majority of academic work investigating medicine in art and entertainment stems from medical dramas. The idea of utilizing television medical dramas as a novel teaching tool has been postulated and discussed through many lenses, but few studies have actually tested the idea in practice. The following were evaluated in course content using television shows: teaching students how to break bad news and deal with difficult situations [14]; and professionalism, including teamwork and communication skills [15, 16]. Ethics was a main topic of teaching for many studies [6, 7, 17], with a focus on psychiatry/mental health in several studies, including teaching psychotherapy [14, 18]. Empathy was the topic of many studies [19–23]. Doctor-patient communication or interactions was also examined [21, 24].

In the papers reviewed regarding the use of movies in medical education, psychiatry/mental health [25–32] dominated as educational topics. Hollywood movies, documentaries, and short films were screened in full or shown as clips to teach students on topics such as professionalism [21, 22, 33], ethics [34–36], cultural competence [5, 37, 38], managing severe or terminal disease [23, 24], breaking bad news [14], death and dying [24], and alcohol and other drugs [39, 40]. The subjects of sexuality [41] and clinical pharmacology [42] were raised in select studies. Specific disease topics mentioned were cancer [24], epilepsy [29], and AIDS [23]. For details about each study,

### Pedagogy/Format (How?)

The articles described a range of pedagogical approaches. While interventions and curricular designs were generally described with limited detail, most articles reported using television and movies to spark discussion. Students either watched television shows and movies in full or viewed a relevant clip. In some cases, the media presentation was followed by a lecture/seminar on the topic and a subsequent discussion in a large group format. Many studies reported on students’ forming small groups for problem-based or case-based learning. In a few instances, students completed reflective essays or narratives. In some, students were asked to conduct presentations or role-play. A question and answer session with the producers of the film, a multiple-choice format to stimulate discussion and recordings of therapy sessions were each used in single studies. Many studies reported their findings as independent, stand-alone educational events.

### Outcomes and evaluation

There were three main types of articles: (1) commentary/perspective encompassing editorials, letters, and general summaries (guides) for television or movies to use for specific topics, which can be quite detailed, as in Hyler and Schanzer’s (1997) guide to 33 movies that depict borderline personality disorder [28]; (2) course descriptions, which describe courses/seminars/workshops, but may not provide specific details about implementation or participants and in which formal evaluation (if any) is not reported; and (3) research or evaluation with clear study design description and outcomes (e.g., literature or systematic review, randomized controlled trials, pre-and post- or pre/post surveys, questionnaires, or tests).

Across our dataset, there were 35 perspective, 43 description, and 35 research/evaluation articles and, overall, most articles were in support of using television and movies in medical education. The studies reviewed suggest television and media use in medical education serves a number of purposes, namely knowledge attainment, skills acquisition, learning enrichment, interest and engagement, debate and discussion, reflection, emotion provocation, and memory retention. The research or evaluation studies examined short-term outcomes and tended to measure student satisfaction and knowledge and, to a lesser extent, attitudes (or some combination). Studies used evaluation methods consistent with Kirkpatrick levels 1 ‘reaction’ (satisfaction) and 2 ‘learning’ (change in attitudes, knowledge).

All studies measuring satisfaction indicated that students were satisfied with the use of television and movies for the purposes of medical education. When knowledge on a subject was evaluated before and after viewing clips, students subjectively reported that the use of entertainment added to their knowledge base. Of the 113 articles abstracted, only three argued against using entertainment media as a teaching tool in medical education [43–45]. The remaining 110 articles reiterated findings from previous studies and commented on the potential to use media clips in the classroom. Those articles not in favour of this teaching methodology expressed concern that ethical encounters and mental health patients were not accurately portrayed in television shows and, therefore, the use of these media to generate discussion on the subject was inherently flawed [43–45].

## Discussion

The use of various media in teaching to address a range of outcomes is not new. In a paper on how video clips embedded in multimedia presentations (including television and movies) can be used to improve learning in college courses, Berk (2009) proposed that the use of clips can attain 20 specific learning outcomes, such as gaining students’ attention, focusing concentration, fostering creativity, stimulating the flow of ideas, making learning fun and decreasing anxiety and tension on upsetting topics, among others [46]. By employing video clips that tap into multiple intelligences and a ‘pluralistic view of the mind’, faculty expose students to a range of learning strategies utilizing verbal, visual and auditory stimuli [46].

In developing their medical expertise and intrinsic competencies (i.e., communication, collaboration, professionalism), medical students are required to master a range of topics. The papers we reviewed highlight an array of subjects covered by this teaching modality, suggesting broad application of this instructional tool across topic areas. Empathy, the focus of many studies [19–23], was the most frequently examined component underlying a person-centred and humanistic approach to medicine. This suggests the importance of this skill and would certainly play a role in doctor-patient communication or interactions, a similarly studied topic [21, 24].

This review found that a range of pedagogical techniques are applied, including watching television shows and movies in full or a relevant clip, accompanied by a large group discussion, small group sessions for problem-based or case-based learning, reflective essays or narratives, presentations or role-play. The varied use of television and movies in medical education may imply that no single pedagogical technique will achieve 100 % effectiveness across all learning styles and all competencies a student is expected to attain. Therefore, television and movies need to be employed with careful consideration of the learning objectives expected to be achieved and the intended outcomes, lest they take time away from better proven educational approaches.

There are simultaneous calls for evidence-informed education and a greater emphasis on a person-centred, humanistic approach to medicine. Therefore, building on the studies we reviewed, there is a need to study approaches that foster competencies associated with the intrinsic roles of humanistic clinicians. Some have advocated efforts to help learners move beyond rote or surface performance of desirable ‘knowledge, skills, and attitudes’ toward a genuine critical consciousness, an embodiment of virtues, and an ability to recognize power differentials, and patients, as individuals influenced by complex life histories and social structures [4, 47]. Teaching related to these topics may benefit from thoughtful applications of pedagogical innovations such as television and movies.

Findings suggest that utilizing television and movies to evoke emotions in trainees stimulates their own emotional awareness thus allowing them to be more empathic with patients. Television and movies are said to heighten learners’ understanding of situational ethics and human suffering [34] and to help them to reflect upon the humanitarian (emotional and psychological) aspects of medicine [48]. As an effective teaching tool, television and movies seem to evoke emotions that stimulate learning about the moral and emotional issues that are part of practising medicine [49]. By appealing to the affective domain of learning, this pedagogical tool may serve to develop trainees’ awareness of their own emotions and empathy, subsequently permitting a better understanding of how to offer emotional support to patients.

A few areas of improvement are needed in future studies assessing the effectiveness of television and movies in medical education. Aside from utilizing post-session evaluation, no study has compared the use of entertainment in the classroom to the standard pedagogical method (i.e., lectures, small groups). Assessing whether or not students actually prefer the use of entertainment to traditional teaching methods, or whether such practices result in an improvement in course performance would be worthwhile.

When implementing such methods, it is important to consider long-term evaluation that goes beyond measuring satisfaction, attitudes and self-perceived knowledge to address Kirkpatrick’s levels 3 (behaviour) and 4 (results). This means measuring how knowledge and skill acquisition translates into behaviour change (clinical application) and thus improved results (patient care). For example, do medical students behave more empathically towards patients?

From a research and evaluation point of view, further studies are needed with richer descriptions of innovations and more rigorous research designs. Additionally, these studies need to fully describe how educational events are incorporated or integrated into the full curriculum. Inadequate detail of such innovations and their evaluation makes it difficult to truly understand what has taken place, and thus to make an informed judgment about their value [50]. Ensuring that an intervention is fully and unambiguously described will encourage curriculum developers to engage in evidence-based curriculum design and employ a sound theoretical framework. Using television and movies in medical education, like eLearning, should not be done solely for novelty. For such innovations to be effective, there needs to be an identified learning gap, a defined theory and a curriculum design that utilizes technology, if appropriate, to address the gap.

Furthermore, given its powerful cognitive and emotional impact, media is a potentially valid approach to tap into the multiple intelligences and learning styles of the ‘net generation’ [46]. The caveat is that, as technology becomes ever more pervasive in society, and as use of emerging media tools (e.g., social media) becomes increasingly widespread, there is greater pressure on faculty to integrate media into their teaching. With such emphasis, careful attention is required to ensure that faculty are adequately prepared to use this instructional tool to deliver quality educational opportunities to medical students.

## Conclusion

There is promise for television and movies to play a unique role in medical education in relation to fostering compassionate, critically conscious care orientations. Some evidence suggests that students find this form of teaching engaging, but further work is needed to justify and facilitate the implementation of this teaching format into curricula, including adequate preparation of faculty. Because a visual form of entertainment is readily available and well-inscribed into popular culture [7], the student population can easily accept and enjoy the use of television and movies throughout their medical education. But if we are to strive toward evidence-informed education, we need an evidence base on this topic to move beyond ‘show and tell’ toward meaningful implementation and evaluation. Further exploration regarding the theoretical basis for using television and movies in medical education will help substantiate continued efforts to use these media as teaching tools.

### Declaration of Interest

The authors report no declarations of interest and no sources of support.

## Electronic supplementary material

(DOCX 45KB)
