# Erratum to: The current landscape of television and movies in medical education

**DOI:** 10.1007/s40037-015-0235-3

**Published:** 2015-11-30

**Authors:** Marcus Law, Wilson Kwong, Farah Friesen, Paula Veinot, Stella L. Ng

**Affiliations:** 1 Department of Family & Community Medicine, Faculty of Medicine, University of Toronto; Centre for Faculty Development, Li Ka Shing Knowledge Institute, Toronto; and Toronto East General Hospital, Room 2325, Medical Sciences Building, 1 King’s College Circle, M5S 1A8 Toronto, ON Canada; 2 Queen’s University, Kingston, ON Canada; 3 Centre for Faculty Development, Li Ka Shing Knowledge Institute, Toronto, ON Canada; 4 Centre for Faculty Development, Li Ka Shing Knowledge Institute, Toronto; Department of Speech-Language Pathology, University of Toronto; Centre for Ambulatory Care Education, Women’s College Hospital, Toronto, ON Canada

**Erratum to**: Law et al. “The current landscape of television and movies in medical education”. Perspect Med Educ (2015) 4(5):218–224

DOI 10.1007/s40037-015-0205-9

Page 221, left column, under the section “Topics/Content (What?)”: Owing to an unfortunate oversight, the last sentence of this section was truncated during proof correction. The sentence should read: “For details about each study, please refer to the Electronic Supplementary Material”.

An extended version of the table in the Electronic Supplementary Material is available with this erratum.

## Electronic supplementary material

(DOCX 135 kb)

