# Supplementary material for: The current landscape of television and movies in medical education
Source: Perspect Med Educ. 2015 Sep 17;4(5):218–24. doi: 10.1007/s40037-015-0205-9 (PMC4602016; doi:10.1007/s40037-015-0205-9)
Supplement: Supplementary file 1 — (DOCX 45KB) [file 40037_2015_205_MOESM1_ESM.docx]

Table 1. Summary of 113 articles

| **RESEARCH – 35 articles** | | | | |
| --- | --- | --- | --- | --- |
| **KIRK-PATRICK** | **INTERVENTION** | **OUTCOME MEASURES** | **CONCLUSIONS** | **ARTICLES (First author, Publication year, Title)** |
| **Level 1**  **Satisfaction (7 articles)** | - Book/Movie Club with discussion - Interactive sessions with combination of TV/movie (in entirety or clips), lecture, discussion, presentations | - Post-intervention questionnaire - Web-based survey - Evaluation forms with close- and open-ended questions | - Student satisfaction with the use of TV/movies or clips was high overall, viewed this method favourably - Students found TV/movies to be believable, thought provoking, relevant to practice, enhanced understanding of humanistic aspects of medicine, captured attention, and provided emotionally engaging experiences | **Alexander** (1994) Cinemeducation: an innovative approach to teaching psychosocial medical care. **Bhagar** (2005) Should cinema be used for medical student education in psychiatry? **Czarny** (2008) Medical and Nursing Students' Television Viewing Habits: Potential Implications for Bioethics. **Lee** (2004) Medical-themed film and literature course for premedical students. **Shevell** (2014) Teaching professionalism to first year medical students using video clips. **Tarsitani** (2004) Cinematic clinical psychiatric cases in graduate medical education. **Wadhwa** (2013) Synoptic philosophy in a neurosurgical residency: a book and cinema club. |
| **Level 2 Learning**  **(4 articles)** | - Movie clips interspersed with survey questions - Seminars with TV medical drama clips - Lecture with movie - Combination lecture, presentation, discussion, TV/movie (in entirety or clips) - Movie clips chosen to highlight empathic understanding in patient encounters with discussion | - Post-intervention survey - 7-item pre/post- seminar surveys - Pre/post/1 month after intervention questionnaire - Jefferson Scale of Empathy (JSE) pre/post and post intervention | - Viewing movie clips influenced attitudes and knowledge about ECT - Knowledge on certain topics improved significantly (countertransference, importance & establishment of patient boundaries, how to break bad news), but not others (establishing boundaries with difficult patients) - Familiar medical dramas have potential for ongoing and reinforcement of learning | **Altindag** (2006) Effects of an antistigma program on medical students’ attitudes towards people with schizophrenia. **Hojat** (2013) Enhancing and sustaining empathy in medical students. **McNeilly** (2001) The ‘ER’ Seminar: Teaching Psychotherapeutic Techniques to Medical Students. **Walter** (2002) Medical Student Knowledge and Attitudes Regarding ECT Prior to and After Viewing ECT Scenes from Movies. |
| **Level 1 + 2 (18 articles)** | - TV/movie (in entirety or clips), viewed during session or on students’ own time - Combination lecture, readings, discussion, presentation to class, role play, reflective writing, small-group/think-pair share | - Pre/post or post- intervention questionnaires - Close-ended questions on Likert scale, multiple choice questions, and/or open-ended qualitative questions - One study (Gallagher) used post-course survey with field notes from student group discussions - Sexual Knowledge and Attitude Test (2^nd^ ed) | - TV/movies are powerful education tool, especially to emphasize social, cultural, and historical contexts of mental illness - Students found TV/movies to be an enjoyable way to learn and contributed to reflection on course materials/topics - Instructors found TV/movies as appropriate for medical education, encouraged independent & collaborative learning, promoted discussion, engaged students successfully, provided safe space for exploration of topics - Objective (instead of self-reported) assessment of knowledge needed | **Aboul-Fotouh** (2010) Therapy 101: a psychotherapy curriculum for medical students. **Datta** (2009) Madness and the movies: an undergraduate module for medical students. **Gallagher** (2014) The efficient use of movies in a crowded curriculum. **Graf** (2014) Development, implementation, and evaluation of a movie-based curriculum to teach psychopathology. **Klemenc-Ketis** (2011) Using movies to teach professionalism to medical students. **Kuhnigk** (2012) Cinemeducation in psychiatry: a seminar in undergraduate medical education combining a movie, lecture, and patient interview. **Lumlertgul** (2009) Cinemeducation: A pilot student project using movies to help students learn medical professionalism. **Mandel** (1983) Medical education about human sexuality: the impact of film in the workshop setting. **Muñoz-Crego** (2009) The teaching of clinical microbiology at the University of Santiago de Compostela by means of film. **Ozcakir** (2014) Educating Medical Students about the Personal Meaning of Terminal Illness Using the Film, ‘Wit.’ **Pais de Lacerda** (2005) Medical education: addiction and the cinema (drugs and gambling as a search for happiness). **Rabow** (2010) Filming the family: a documentary film to educate clinicians about family caregivers of patients with brain tumours. **Retamero** (2014) Use of the Film The Bridge to augment the suicide curriculum in undergraduate medical education. **Ross** (2011) Using film in multicultural and social justice faculty development: scenes from Crash. **Self** (1993) Teaching medical ethics to first-year students by using film discussion to develop their moral reasoning. **Sierles** (2005) Using film as the basis of an American culture course for first-year psychiatry residents. **Welsh** (2003) ODs and D's: using movies to teach intoxication and withdrawal syndromes to medical students. **Wong** (2009) Using television shows to teach communication skills in internal medicine residency. |
| **N/A**  **(6 articles)** | - The articles were categorized as research, but without Kirkpatrick levels, due to the fact that they are reviews - Systematic review of literature (Darbyshire) - Qualitative review of TV medical dramas with thematic analysis (Czarny) - Comprehensive, annotated reviews of movies (Castaldelli-Maia, Flores 2002, Flores 2004, Wilson) | - Systematic review of literature summarized topic areas that have used movies in medical education and identified gaps for future study - Qualitative review of TV programs assessed content available on bioethics/ professionalism in medical dramas - Comprehensive, annotated reviews of movies serve as guides to help educators choose appropriate movies for teaching certain topics | - Systematic review of literature showed need for more descriptive accounts of pedagogy to build evidence base for efficacy of movies in medical education - Qualitative review of TV medical dramas found programming rife with bioethical issues and deviations from professionalism, with only few exemplary depictions of professionalism, but could help discussion about ethical/professional handling of issues portrayed - Suggest future research on the effect of medical dramas on attitudes/behaviour of patients & healthcare professionals and on evaluation of medical students’ self-directed learning by viewing movies | **Castaldelli-Maia** (2012) Using selected scenes from Brazilian films to teach about substance use disorders, within medical education. **Czarny** (2010) Bioethics and professionalism in popular television medical dramas. **Darbyshire** (2012) A systematic review and thematic analysis of cinema in medical education. **Flores** (2002) Mad scientists, compassionate healers, and greedy egotists: The portrayal of physicians in the movies. **Flores** (2004) Doctors in the movies. **Wilson** (2014) Madness at the movies: prioritised movies for self-directed learning by medical students. |
| **DESCRIPTIVE – 43 articles** | | | | |
| **KIRK-PATRICK** | **INTERVENTION** | **OUTCOME MEASURES** | **CONCLUSIONS** | **ARTICLES (First author, Publication year, Title)** |
| **Level 1**  **Satisfaction**  **(12 articles)** | - Elective courses, special study modules, or workshops - Small group work or guided private study - Combination lecture, discussions, readings, TV/movies (in entirety or clips), essays, creative writing, facilitated, and panels (e.g. commentators, movie critics, psychiatrist) | - ‘Descriptive’ articles do not provide clear reporting of ***how*** outcomes were assessed or measured, even though sample student quotes are provided - Unclear how quotes were gathered, student satisfaction assessed, etc. except in some cases when authors state ‘informal feedback was gathered’ - One post-presentation survey evaluating use of clips (Pavlov), but survey questions and scale not described, only that all rated 4 or 5 with 5 representing ‘extremely helpful’ to stimulate interest | - Student satisfaction with the use of TV/movies in entirety or clips was high overall - Lively, enthusiastic participation in discussions by students, with many of the seminars/workshops extended in following years - Authors found movies engaged learners emotionally while fostering reflection on certain topics (e.g. psychiatry) and attitudes/values (e.g. humanitarian, emotional, psychological aspects of medicine, empathic attitudes, and professional values - Suggest future research evaluating whether or not the use of TV/movies increases knowledge (e.g. pre/post surveys to document objective effectiveness of TV/movies to teach specific topics) | **Akram** (2009) Crossing the line--learning psychiatry at the movies. **Alexander** (2000) Cinemeducation: Teaching family systems through the movies. **Blasco** (2001) Literature and movies for medical students. **Blasco** (2010) Cinema for educating global doctors: From emotions to reflection, approaching the complexity of the human being. **Blasco** (2010) Teaching through movies in a multicultural scenario: Overcoming cultural barriers through emotions and reflection. **Elder** (2002) Using the cinema to understand the family of the alcoholic. **Glasser** (2001) From Kafka to Casualty: Doctors and medicine in popular culture and the arts - A special studies module. **Johnson** (2014) Using Breaking Bad to teach about defence mechanisms. **Jukić** (2010) Movies in education of psychiatry residents. **Lepicard** (2003) Medicine, cinema and culture: A workshop in medical humanities for clinical years. **Loscos** (2006) Medicine, cinema and literature: a teaching experiment at the Universitat Autonoma de Barcelona. **Pavlov** (2010) Teaching communication and professionalism using a popular medical drama. |
| **Level 1+2 (7 articles)** | - Monthly or weekly seminar, movie clubs - Combination movies (popular films and documentaries, in entirety or clips), facilitated discussions, readings, guest speakers, reflection sheets, essays, and role-play | - ‘Descriptive’ articles do not provide clear reporting of ***how*** outcomes were assessed or measured, even though sample student quotes are provided - Only one article (Goldman) details use of post-seminar questionnaire with 11 Likert-type (5-point) questions + 1 open-ended question (additional comments) on material covered, organization, teaching | - Student satisfaction with the use of movies was high overall (authors report this based on comments such as ‘thought-provoking,’ ‘memorable,’ ‘great medium to teach’) - When movies watched in entirety, students connected with characters & come to see patients as complex people - Movies broadened perspectives to include humanistic, social, philosophical levels and stimulated creative thinking - Movies helped students develop empathy for patients and increased knowledge of certain topics (e.g. unconscious dynamics in psychiatry, Asperger’s, stigma of mental health, identifying IPV and intervention strategies, illness’ impact on patients/families, child psychiatry, comprehensive approach to complex disease, cultural/religious awareness) | **Fritz** (1979) The role of a cinema seminar in psychiatric education. **Goldman** (1987) An elective seminar to teach first-year students the social and medical aspects of AIDS. **Gorring** (2014) Cinemeducation: Using film as an educational tool in mental health services. **Lenahan** (2005) Facilitating the emotional education of medical students: Using literature and film in training about intimate partner violence. **Murphy-Shigematsu** (2010) The impact of film in teaching cultural medicine. **Weber** (2007) Movies and medicine: An elective using film to reflect on the patient, family, and illness. **Zerby** (2005) Using the science fiction film invaders from mars in a child psychiatry seminar. |
| **N/A**  **(24 articles)** | - Programs, seminars, elective modules, movie clubs - Combination TV/movies (in entirety or clips), facilitated discussions, readings, lecture, case presentations, creative writing (movie scripts of breaking bad news encounters), final exam | - These articles were not assigned Kirkpatrick levels due to the fact that they are descriptions of proposed or previous programs, but no study data is reported (e.g. number of participants, how outcomes assessed, etc.) - Articles are useful guides to TV/movie themes/synoses, with detailed discussion points and learning objectives - One article (Rieder) provides exam questions, though does not report on any results - 1 literature review (Lim RF) | - Although no detailed study data provided, authors have used TV/movies in their own teaching and describe their use of the intervention - Authors conclude that TV/movies are effective at generating discussion and encouraging learning, though no objective evaluation is provided - One article (Kalra 2011) cautions that movies are made for entertainment, not education, so can present only individual writers’/directors’ perspectives about a topic (e.g. mental illness) and do not necessarily present scientific understanding - Care should be exercised in choosing TV/movies so as not to perpetuate stereotypes - Author (Alexander 2006) suggests more work on whether students prefer/value this type of learning more than traditional forms of learning, but with direct observation/questionnaire to assess knowledge | **Alexander** (2006) What’s Eating Gilbert Grape?: A case study of chronic illness. **Belling** (2006) The ‘bad news scene’ as clinical drama part 1: writing scenes. **Belling** (2006) The ‘bad news scene’ as clinical drama part 2: viewing scenes. **Blasco** (2006) Using movie clips to foster learners’ reflection: Improving education in the affective domain. **Cape** (2009) Movies as a vehicle to teach addiction medicine. **Crellin** (1995) Movies in medical education. **Desai** (2014) Alternate methods to teach history of anesthesia. **Farré** (2013) Bioethical principles, clinical research and popular movies. **Fresnadillo Martínez** (2005) Teaching methodology for the utilization of cinema in the teaching of medical microbiology and infectious diseases. **Gramaglia** (2013) Cinema in the training of psychiatry residents: focus on helping relationships. **Henry** (2009) Movies and the medical profession. **Kalra** (2011) Psychiatry movie club: A novel way to teach psychiatry. **Kalra** (2012) Talking about stigma towards mental health professionals with psychiatry trainees: A movie club approach. **Kaye** (2000) The Breakfast Club: Utilizing popular film to teach adolescent development. **Koren** (1993) Awakenings: using a popular movie to teach clinical pharmacology. **Lim RF** (2008) Using non-feature films to teach diversity, cultural competence, and the DSM-IV-TR outline for cultural formulation. **Lim EC** (2008) In-house medical education: redefining tele-education. **Misch** (2000) Psychosocial formulation training using commercial films. **Quadrelli** (2009) Appreciation of the aesthetic: A new dimension for a medicine and movies program.  **Rieder** (1974) Teaching about schizophrenia.  **Self** (1990) Teaching medical humanities through film discussions. **Tobia** (2013) The horror!: A creative framework for teaching psychopathology via metaphorical analyses of horror films. **Winter** (2005) Family systems at the movies. **Yamada** (2003) Cross-cultural ethics and the moral development of physicians: Lessons from Kurosawa’s Ikiru. |
| **PERSPECTIVE – 35 articles** | | | | |
| **KIRK-PATRICK** | **INTERVENTION** | **OUTCOME MEASURES** | **CONCLUSIONS** | **ARTICLES (First author, Publication year, Title)** |
| **N/A**  **(35 articles)** | - ‘Perspective’ are commentaries or editorials - Articles provide authors’ perspectives on the usefulness of TV/movies | - N/A - articles in this category are commentaries or editorials, therefore no study data. | - Authors describe their reasons for supporting this intervention, highlighting educational value based on previous experiences - Suggestions of suitable TV/movies, detailed learning objectives, and discussion points serve as guides for other educators - 3 articles (Greenberg, Ramchandani, Trachtman) caution against the use of TV/movies due to possible inaccuracies, misrepresentation, or misleading ‘easy’ solutions common to fictional representations of complex situations - Suggest future research testing hypothesis that teaching points using TV/movies are more readily understood and maintained than by other approaches | **Alexander** (2002) The doctor: A seminal video for cinemeducation. **Alexander** (2007) Lights, camera, action: Using film to teach the ACGME competencies. **Alexander** (2009) The couple’s odyssey: Hollywood's take on love relationships. **Arawi** (2010) Using medical drama to teach biomedical ethics to medical students. **Astudillo Alarcón** (2007) The cinema in the teaching of medicine: Palliative care and bioethics. **Baños** (2007) How literature and popular movies can help in medical education: Applications for teaching the doctor-patient relationship. **Baxendale** (2011) Wrestling fact from fiction. **Bhugra** (2003) Using film and literature for cultural competence training. **Bhugra** (2003) Teaching psychiatry through cinema. **Cappelletti** (2007) Can we teach better? The relationship between the cinema and teaching. **Czarny** (2008) Response to open peer commentaries on ‘Medical and nursing students’ television viewing habits: potential implications for bioethics.’ **Dave** (2011) Cinemeducation in psychiatry. **Dobson** (2004) Can medical students learn empathy at the movies? **Farré** (2004) Putting clinical pharmacology in context: the use of popular movies. **Furst** (2007) Bowlby goes to the movies: Film as a teaching tool for issues of bereavement, mourning, and grief in medical education. **Greenberg** (2009) Caveat actor, Caveat emptor: some notes on some hazards of Tinseltown teaching. **Guerrero** (2007) The fall and redemption of people and systems: Potential lessons from the ‘Star Wars’ saga. **Halamek** (2010) Lost moon, saved lives: Using the movie Apollo 13 as a video primer in behavioural skills for simulation trainees and instructors. **Hallberg** (2007) 8 films medical students should see. **Hirt** (2013) Medical dramas on television: a brief guide for educators. **Hyler** (1997) Using commercially available films to teach about borderline personality disorder. **Kalra** (2011) Teaching diagnostic approach to a patient through cinema. **Midmer** (2004) Cine-ed: Using films to teach medical learners. **Miller** (1999) Using the movie Ordinary People to teach psychodynamic psychotherapy with adolescents. **Mischoulon** (2004) ‘The Matrix’: An allegory of the psychoanalytic journey. **Raballo** (2009) Humanizing the clinical gaze: Movies and the empathic understanding of psychosis. **Ramchandani** (2012) The downside of teaching psychopathology with film. **Rosenstock** (2003) Beyond a beautiful mind: Film choices for teaching schizophrenia. **Sondheimer** (2000) The life stories of children and adolescents: Using commercial films as teaching aids. **Spike** (2008) Television viewing and ethical reasoning: why watching Scrubs does a better job than most bioethics classes. **Trachtman** (2008) The medium is not the message. **Tsai** (2013) Movie dialogues as discourse data in the study of forecasting mechanisms in the delivery of medical bad news. **Volandes** (2007) Medical ethics on film: Towards a reconstruction of the teaching of healthcare professionals. **White** (2008) Capturing the ethics education value of television medical dramas. **Wicclair** (2008) The pedagogical value of House, M.D.--can a fictional unethical physician be used to teach ethics? |
