# Supplementary material for: Erratum to: The current landscape of television and movies in medical education
Source: Perspect Med Educ. 2015 Nov 30;4(6):352. doi: 10.1007/s40037-015-0235-3 (PMC4673074; doi:10.1007/s40037-015-0235-3)
Supplement: Supplementary file 1 — (DOCX 135 kb) [file 40037_2015_235_MOESM1_ESM.docx]

**Table 1: Summary of 113 Articles**

| **Article**  (alphabetical by author) | **Purpose of intervention** | **Study Design** | **Type of paper**/ **Participant or Target Population** | **Outcomes/**  **Variables of interest** | **Results/Conclusions** | **Kirkpatrick Levels** |
| --- | --- | --- | --- | --- | --- | --- |
| Aboul-Fotouh F, Asghar AA. Therapy 101: A psychotherapy curriculum for medical students. Acad Psychiatry. 2010;34:248–52. | To introduce medical students to basic understanding of and the practice of psychotherapy, including recognizing the relevance of psychotherapy principles to the doctor-patient relationship, awareness of psychotherapies with evidence-based efficacy for particular disorders, and be able to refer patients for psychotherapy. | Third year medical students (n=8) participated in "Therapy 101: An Introduction to Psychotherapy," a 6-hour curriculum with creative teaching techniques such as video clips from TV shows, role play, and recordings of therapy sessions. The series was evaluated using a multiple-choice pre/postcourse test with questions on psychotherapy, written feedback, and perceived knowledge of psychotherapy. | Research  3rd year medical students | Student knowledge of psychotherapy through pre/postcourse test and student self-rating of knowledge. Student satisfaction with the course. | The scores from the pre/postcourse test measuring the students' knowledge of psychotherapy did not change significantly. However, students' self-rating of knowledge greatly increased after the series. Written feedback showed substantial enthusiasm for the course. The mean number of questions answered correctly was 11.3/15 on the multiple-choice precourse test and 11.6/15 on the postcourse test. The mean for the students' rating of their current knowledge of psychotherapy at the onset of the course was 3.4 (1=no knowledge, 10=extremely knowledgeable). By the last session, this rating had increased to 6. Students can benefit from a short and engaging curriculum on psychotherapy. "Therapy 101" was very well accepted by students and has been integrated into the curriculum for students in the mental health pathway. | 1, 2 |
| Akram A, O’Brien A, O’Neill A, Latham R. Crossing the line--learning psychiatry at the movies. Int Rev Psychiatry. 2009;21(3):267–8. | To use specific movies to direct medical students in learning about specific psychiatric disorders, how to interact with mental health patients, and uncover common misassumptions. Movies can overcome constraints of demonstrating psychiatric conditions in safe and ethical way. | Brief description of Special Study Module (SSM), which is in addition to core curriculum, offers in-depth study on a topic (Psychiatry and Film). Students view films, discuss, and write an essay summarizing experience and make 5 minute presentation to class. | Description  Medical students | Qualitative comment from students about what they learned from the SSM on Psychiatry and Film, and whether they enjoyed the experience. | No description of how they evaluated/assessed outcome. One student quote is provided as example of feedback. Quote provided states "every student who participated in the SSM spoke extremely highly of it." Authors state the SSM provokes interest in psychiatry as medical specialty and movies are a positive tool for education and generating lively debate. | 1 |
| Alexander M. The doctor: A seminal video for cinemeducation. Fam Med. 2002;34(2):92–4. | Describes author's experience using the movie *The Doctor* to teach 1) interviewing skills, 2) delivering bad news, 3) the psychosocial impact of terminal illness, 4) balancing work and home, 5) cross-cultural issues in medicine, 6) hospital bureaucracy and patient satisfaction, 7) legal issues in medicine, 8) gender and medicine, and 9) effective residency education. | N/A (not a study). Author provides summary of movie clips from The Doctor, sample discussion questions, and role-plays useful for teaching the first 4 topic areas listed. | Perspective  Guide for educators, but suitable for learners | N/A | Author states no outcome research yet conducted to test the hypothesis that teaching points from this technique are more readily understood and maintained than by other approaches. Author's experience is that the approach of movie clips and discussion is successful with residents and medical students, but no actual study or data. | N/A |
| Alexander M. The couple’s odyssey: Hollywood's take on love relationships. Int Rev sychiatry (Abingdon, England). 2009;21(3):183–8. | To present author's description of uses of movies for to teach couple issues to psychiatry residents (eg. Attachment, de-escalation, conflict resolution, common problem areas, etc). Author provides summary of numerous movie clips and sample discussion questions. | N/A (not a study) | Perspective  Guide for educators, but suitable for learners | N/A | N/A | N/A |
| Alexander M, Hall MN, Pettice YJ. Cinemeducation: an innovative approach to teaching psychosocial medical care. Fam Med. 1994;26(7):430-3. | To teach psychosocial aspects of medicine (including DSM diagnosis) using movie clips. | Incorporated movies clips into 1-hour teaching sessions to engage residents' reflection on patients from clinical practice, illustrate family life cycle issues, and Diagnostic and Statistical Manual of Mental Disorders-Revised diagnoses. | Research  Family medicine residents | Student satisfaction. | Author concludes that movies have the ability to capture learners' attention, expose residents to diverse lifestyles, engage the humanistic side of physicians, and imprint powerful pictorial images in memory. Teaching with movie clips is time efficient and provides emotionally engaging experiences for both faculty and residents. | 1 |
| Alexander M, Pavlov A, Lenahan P. Lights, camera, action: Using film to teach the ACGME competencies. Fam Med. 2007;39(1):20–3. | To use "cinemeducation" (movie clips or full-length movies) as a tool to facilitate the teaching of Accreditation Council for Graduate Medical Education (ACGME) competencies as part of an overall family medicine curriculum. Movies are suggested for each of the 6 ACGME competencies with guided discussion, role play, and lecture ideas. | N/A (not a study) | Perspective  Guide for educators, but suitable for learners | N/A | No actual study or data. Authors suggest 3 books on cinemeducation for further information on using popular movies as an innovative, fun, informative teaching tool for ACGME competencies. Authors state current evidence for use of cinemeducation is mostly anecdotal and look forward to further research on the subject. | N/A |
| Alexander M, Waxman D. Cinemeducation: Teaching family systems through the movies. Fam Syst Heal. 2000;18(4):455–66. | To describe a teaching methodology where movies were used to teach medical students about complex family systems and different dynamics within families. | Description of teaching sessions used with different student populations. Session starts with movie clips to illustrate a teaching point or family systems concept, followed by facilitated discussion. Details provided for movie clips with different themes including family genogram, differentiation, homeostasis, over/underfunctioning, cross cultural coupling, and initiating/leading family conference. | Description  3rd year medical students  2nd year family medicine residents | No outcomes/evaluation assessed or reported. | No formal evaluation, but authors got very good verbal feedback from students in terms of how much they enjoyed the program ("consistently receive enthusiastic verbal feedback about their enjoyment and long-term recall of the clips.") Formal research evaluating whether or not this method increases knowledge is encouraged. | 1 |
| Alexander M, Waxman D, White P. What’s Eating Gilbert Grape?: A case study of chronic illness. J Learn Through Arts. 2006;2(1), article 13. | To describe the use of a movie (*What's Eating Gilbert Grape)* to teach medical students about the biopsychosocialspiritual approach to chronic illness. | As part of 3rd year medical students' 4-week clerkship in family medicine, authors teach biopsychosocialspiritual approach to chronic illness. Session format: 3 hours long, 1 day/week, requirements for chronic illness presentation are reviewed, discussion about chronic and acute illness differences, brief lecture contrasting biomedical versus biopsychosocialspiritual model, "Gilbert" is introduced as patient seeing physician repeatedly for low back pain with little resolution, and screening of opening clip of movie. This introduces students to genograms and family circles. At the end of the last session, educators present an exemplary presentation of chronic back pain (using opening movie clip) to students (detailed case provided in the article). | Description  Family practice residents | No formal evaluation or assessment conducted. | Authors report subjective observations that the use of the movie is rated highly by students (no data presented), but also state that more work is needed to actually see if students prefer/value this type of learning more than traditional forms of learning and with direct observation or questionnaire to assess student knowledge. | N/A |
| Altindag A, Yanik M, Ucok A, Alptekin K, Ozkan M. Effects of an antistigma program on medical students’ attitudes towards people with schizophrenia. Psychiatry Clin Neurosci. 2006;60:283–8. | To examine whether an antistigma program which consists of education, contact, and viewing a movie that depicts an individual with schizophrenia (*A Beautiful Mind)*, can change attitudes towards people with schizophrenia. | Intervention group (n=25) part of 1 day antistigma program received 2 hour lecture, presentation and discussion with a person with schizophrenia, and viewing of *A Beautiful Mind*. Control group (n=35) part of 1 day program received 2 hour lecture about water metabolism was and viewed *Winged Migration* (documentary on migratory patterns of birds). Both intervention and control groups had received no prior education on mental health and psychiatry in the school. Questionnaire with 10 items on demographic/health status and 32 items rating attitudes towards schizophrenia administered pre-, post-, and 1 month after intervention. | Research  1st year medical students | Student attitudes towards and perceptions (beliefs) related to schizophrenia. | Intervention group observed favourable attitudinal changes in 'belief about the etiology of schizophrenia', 'social distance to people with schizophrenia', and 'care and management of people with schizophrenia'. In contrast, no significant change was observed in the control group. Attitude changes tended to decrease at the 1-month follow up. Results suggest that attitudes towards schizophrenia can be favourably changed with the intervention (antistigma program). To sustain changed attitudes towards people with schizophrenia, antistigma programs should be offered on a regular basis. Authors suggest future research to examine whether improved attitude corresponds with change in behavior. | 2 |
| Arawi T. Using medical drama to teach biomedical ethics to medical students. Med Teach. 2010;32:e205–10. | Perspective article where author makes the case that medical drama as narrative genre (md-narrative) enhances emotional engagement, cognitive development, and moral imagination which allow for a more ethically sensitive student in training. Author posits that case vignettes are not enough and can objectify and render patients soulless, whereas medical drama can bring patients to life. Uses House MD as detailed example. | N/A (not a study). | Perspective  Paper for educators, but suitable for learners. | N/A | Author mentions preparing pilot program with MED III students at the American University of Beirut teaching hospital in the Physicians, Patients and Society-2 course. House MD will be used in lieu of clinical vignettes. Some will be screened in class and interrupted on the go for questions, debates, and reflection. Others will be divided into segments and placed online via a Learning Management System (Moodle) and be used as entrance and exit activities for a number of units. Data will see whether md-narratives contribute to students' emotional and ethical maturity. | N/A |
| Astudillo Alarcón W, Mendinueta Aguirre C. The cinema in the teaching of medicine: Palliative care and bioethics. J Med Movies. 2007;1:32–41. | Authors posit that movies can be used to teach palliative care topics (eg. disease, loneliness, death, suicide, grief) and bioethics (eg. confidentiality and patient autonomy). Authors provide lists of movies with detailed description of the relevant palliative care or bioethics themes in select movies. | N/A | Perspective  Guide for educators, but suitable for learners | N/A | N/A | N/A |
| Baños JE. How literature and popular movies can help in medical education: Applications for teaching the doctor-patient relationship. Med Educ. 2007;41(9):918. | To outline how medical students can benefit from watching 2 specific movies, *The Case of Dr. Sachs* and *The Doctor* (or the books that they were adapted from) as the movies document how doctors interact with patients in a favourable way when breaking bad news or managing someone with a very severe disease. | N/A (not a study) | Perspective  Guide for educators, but suitable for learners | N/A | N/A | N/A |
| Baxendale S. Wrestling fact from fiction. Epilepsy Behav. 2011;22(3):420. | Editorial about how the movie *Stigmata* (other specific movie clips) can be used to teach students about epilepsy. Specifically, *Stigmata* has potential to teach differential diagnosis of epilepsy. | N/A (not a study) | Perspective  Editorial for educators, but suitable for learners | N/A | N/A | N/A |
| Belling C. The “bad news scene” as clinical drama part 1: Writing scenes. Fam Med. 2006;38(6):390–2. | To prepare students for sharing bad news through understanding how giving bad news is a performance so that they might further understand what makes an appropriate and inappropriate performance. | Medical students were asked to think about a time when they had to give bad news, and then write out a 'movie script' version of that encounter. Afterwards, the class discussed these scripted encounters and even had some students act them out. Two film clips are viewed immediately after discussing the written scenes. | Description  Medical students (4th year) | None directly measured. | Authors posit the usefulness of using movies and discussion to help students reflect upon their role in delivering bad news, but does not go on to evaluate how students reacted and perceived the medicine & film elective. No outcome was assessed/reported. | N/A |
| Belling C. The “bad news scene” as clinical drama part 2: Viewing scenes. Fam Med. 2006;38(7):474–5. | To encourage students to approach the bad news conversation as a scene to develop a necessary distance from themselves and their anxieties, but not from the patient and family. Viewing themselves as part of the patient’s drama with a role to perform can help students take responsibility for making the "bad news" turning point as positive as possible. | Medical students viewed movie clips and full-length movies where patients were told bad news, followed by group discussion of how both doctors and patients must have felt in the scenarios viewed. | Description  Medical students (4th year) | None directly measured. | Authors posit the usefulness of using movies and discussion to help students reflect upon their role in delivering bad news, but does not go on to evaluate how students reacted and perceived the medicine & film elective. No outcome was assessed/reported. | N/A |
| Bhagar H a. Should cinema be used for medical student education in psychiatry? Med Educ. 2005;39(9):972–3. | To present disorders or diseases in psychiatry as syndromes and introduce the multi-axial diagnostic system with popular movie intervention. Letter in response to and a critique of Tarsitani *et al.*'s results (lacks control group). | Brief description of original course with PowerPoint presentation to discuss diagnosis in depression, schizophrenia and personality disorders (control group). The next year, Bhagar presented the PowerPoint augmented by clips from *The Hours* (suicidal/depressed woman), *A Beautiful Mind* (delusions, auditory/visual hallucinations), and *As Good As It Gets* (compulsive avoidance) (intervention group). | Research  2nd year medical students | 2 questions on the evaluation: (A) were objectives defined; and (B) did the lecture presentation meet objectives? | In control group (2003), 36% of the students submitted evaluations with average rating on question A 3.3, and question B 3.2. In intervention group (2004), 33% of the students returned evaluations with average rating on question A 3.4, and on question B 3.3. The student comments were strongly favourable to the use of the movie clips, but the overall scores both before and after the addition of cinematic scenes were statistically similar. There are several limitations to both surveys and evidence from long-term controlled studies for evaluation of this supplemental teaching aid should be the next step. | 1 |
| Bhugra D. Using film and literature for cultural competence training. Psychiatr Bull. 2003;27(11):427–8. | To share author's perspective on how movies and literature can be used to teach about cultural competence. Author states that novels and films provide portrayals of different cultural norms and conflicts, which can help develop trainees' humanism and capacity for understanding cultural competence. Author shares 14 films suitable for illustrating cultural differences in Asian, African American, Chinese, and Latin cultures. | N/A (not a study) | Perspective  Guide for educators. | N/A | N/A | N/A |
| Bhugra D. Teaching psychiatry through cinema. Psychiatr Bull. 2003;27(11):429–30. | To share author's perspective on movies that can be used to teach psychiatry. Author provides list of 34 movies covering topics of mental state examination, diagnosis, doctor-patient interactions, and personality disorder. Advantage of using movies: realistic depictions of character styles and psychopathologic disorders, as well as personal and family dynamics. Disadvantage of using movies: distortion and stigmatising portrayal of mental illness. | N/A (not a study) | Perspective  Guide for educators. | N/A | N/A | N/A |
| Blasco PG. Literature and movies for medical students. Fam Med. 2001;33(6):426–8. | To learn how to profit from the enthusiasm of medical students for literature and film to help them obtain a deeper and more compassionate understanding of humanity, in preparation for their future roles as doctors. | Brief description of the Academic Department of the Brazilian Society of Family Medicine's (SOBRAMFA) extracurricular educational project, "Literature and Movies for Medical Students." Students pick 1 book and 1 movie to read/watch on their own, then have informal meetings led by students to discuss the movies/books. | Description  1^st^-5^th^ year medical students (n=40) | Students discussed questions about the nature of medicine, sickness & health, life & death, suffering & joy, love, loyalty, compassion, one's motivations for medicine as a profession, physicians' aspirational ideals, and physician qualities sought by patients. | No clear description of how outcome/evaluation assessed (eg. survey or observation). Authors use some student quotes where students reflect on the professional mission of the physician, including physician qualities and whether the technical acquisition of medical knowledge caused the loss of the humane factor in physicians. Authors note that open discussion after movies was a useful and enjoyable way to stimulate reflection on personal and professional attitudes and values of medical students. | 1 |
| Blasco PG, Garcia DSO, de Benedetto MAC, Moreto G, Roncoletta AF, Troll T. Cinema for educating global doctors: From emotions to reflection, approaching the complexity of the human being. Fam Med. 2010;10(3):45–7. | To describe the cinematic teaching methodology using movie clips to promote reflection on attitudes and human values, as delivered to medical educators. | Workshop with more than 100 attendees. Medical educators were shown movie clips in rapid succession (as per authors' methodology) and asked to reflect on specific scenes. These scenes were meant to show them how movies can make them reflect, and were not necessarily medicine related. | Description  Medical educators | No description of formal evaluation or assessment provided, but authors report participant feedback through comments on their satisfaction and reaction to the teaching methodology. | From the comments included by the authors, medical educators agree that movies can help foster reflection. Comments included "pleasant experience," "works as tremendous recall for keeping the focus," and "terrific experience." Authors conclude that teaching with movies can increase re flection, promote empathic attitudes, enrich professional values, and that movies are an innovative resource that can broaden the range of human experience for better understanding of the human being. | 1 |
| Blasco PG, Moreto G, Roncoletta AFT, Levites MR, Janaudis MA. Using movie clips to foster learners’ reflection: Improving education in the affective domain. Fam Med. 2006;38(2):94–6. | To describe the movie clip teaching methodology to foster reflection in learners. | Description of movie clip methodology: 1) appendix of movie clips; 2) instructor comments during clips to facilitate reflection; 3) discussion focusing not only on emotional responses, but reasons for students' feelings, intending to promote reflection on health/illness in relation to human experience, discussion of conflicts/concerns students feel in regards to their professional roles. No details about number of participants, how many sessions were run, etc. | Description  Medical students | No detail on how (if any) assessment was done, but includes student quotes about their experience with the movie clip teaching methodology. | No clear description of how outcome/evaluation assessed, but authors include some participant quotes. Student quote related to movie clips inspiring reflection on their choice of medicine as profession. Authors state measuring outcomes such as sympathy, compassion, and commitment are difficult, but that movie clips are a powerful resource to promote reflective attitudes and to provide learning linked to experience. Authors state that students report (no direct quotes) that movie clip training acts like "an alarm" to make students more aware when issues/situations presented on screen occur in their daily lives. It is not clear how this assessment/feedback was done or how this knowledge change was measured. | N/A |
| Blasco PG, Mônaco CF, De Benedetto MAC, Moreto G, Levites MR. Teaching through movies in a multicultural scenario: Overcoming cultural barriers through emotions and reflection. Fam Med. 2010;42(1):22–4. | To see if cinema-education works in a multicultural scenario to teach reflection through film. The team previously used the approach of teaching with cinema, but only to homogeneous Latin audience. Cinema-education defined as using movie clips to inspire reflection in learners about their attitudes, engaging learners emotionally, not only intellectually. | Workshops for 60-90 participants. Format: Introductions, 20-30 mins movie clips in rapid succession with facilitator comments, and open discussion for reflection, sharing of feelings/thoughts. | Description  Medical students, family medicine residents, faculty | To see whether a non-Latin audience responded in an emotionally engaged and open manner to cinema-education aimed at engaging reflection. | No clear description of how outcome/evaluation assessed, but authors include some participant quotes. Participant quotes include observation that movie clips stimulate more reflection while engaging learners emotionally, have greater educational value than literature classes, and that cinema-education works for a multicultural audience. Authors state that teaching reflection through film is not just watching movies or mastering subject matter, but evoking emotions and considering moral & emotional issues that are part of practicing medicine. | 1 |
| Cape G. Movies as a vehicle to teach addiction medicine. Int Rev Psychiatry. 2009;21(3):213–7. | To describe previous use of movie clips to teach about addiction medicine (alcohol & drug use subspecialty only). To demonstrate how stereotypes of alcohol/drug use in movies may be used to engage, impart information, challenge, and entertain students of addiction medicine. | Describes movie myths related to addiction (eg. recovery with "enough" compassion/love, withdrawal symptoms). Provides example of movies with specific addiction-related details to look for that can be used followed by facilitated discussion to teach addiction and dependence topics to students (*The Lost Weekend, Leaving Las Vegas, Drug Store Cowboy, Pulp Fiction, Withnail and I, Reefer Madness).* | Description  Medical students | No detail on how (if any) assessment was done. | No clear description of how (if any) outcome/evaluation assessed, though authors state they have "tried and tested use of this potent educational aid." Authors conclude that movies have almost universal appeal and therefore have the potential to enhance therapeutic commitment (knowledge, context, support) of addiction medicine. Care should be exercised in choosing movies as an educational tool so as not to perpetuate stereotypical portrayals (myths) about addiction. | N/A |
| Cappelletti GL, Sabelli MJG, Tenutto MA. Can we teach better? The relationship between the cinema and teaching. J. Med. Movies. 2007;3:87–91. | Authors present their reasons for the effectiveness of movies as a teaching tool based on theory (Bruner, Gadamer, Jauss, de la Torre, Roig) and extend this briefly to medicine. No specific movies are mentioned. | N/A | Perspective  Commentary for medical and health professional educators. | N/A | N/A | N/A |
| Castaldelli-Maia J, Pereira Oliveira H, Guerra Andrade A, Lotugo-Neto F, Bhugra D. Using selected scenes from Brazilian films to teach about substance use disorders, within medical education. Sao Paulo Medidcal J. 2012;130:380–91. | A review of Brazilian films to select scenes with potential to teach medical students the main concepts relating to alcohol and drug abuse and dependence, their diagnosis and their impact on others and on society. Each of the reviewers rated how useful these would be in teaching medical students and psychiatry trainees. | Systematically reviewed Brazilian movies made during the Revival of National Cinema from 1994 to 2008. Rating scale developed to determine best films for teaching this subject. On this scale, each relevant scene gained one point, each substance displayed gained one point, each related disorder found in ICD-10 gained one point, police involvement gained one point; and each scene that had a grade > 7.99 in relation to teaching applicability at the end of phase 2 gained two points. | Research (review)  Medical students and psychiatry trainees. | Systematic review and rating of Brazilian movies from 1994-2008 to determine crucial scenes to teach addiction (alcohol and drug abuse). | The review produced a method for appropriately choosing and rating movies relevant to teach addiction. 39 scenes from 27 movies are described with ratings for topics related to alcohol, opioids, cannabis, sedatives/hypnotics, cocaine, hallucinogens, volatile solvents, multiple drugs, and treatment. | N/A |
| Crellin JK, Briones AF. Movies in medical education. Acad. Med. 1995;70:745. | To describe the use of popular films as an innovation to incorporate humanities into medical education to encourage reflection on how the public views the medical profession, how values in movies compare with values of individuals in various stages of medical enculturation. | Brief description of Films in Medical Education (FIME) program at Memorial University. The program consists of series of informal modules include films, narrative, and workbook around a certain theme. | Description  Students & faculty from medicine + related disciplines; community physicians; public. | Module themes include: managing mental illness; procuring and dissecting human bodies for anatomy; societal & medical understanding of disease; culture & sexuality; medical education in movies. | No evaluative data. Description states that the program has increased awareness of the application of popular culture to medicine and enhanced student-faculty dialogue on personal values and approaches to medicine. | N/A |
| Czarny MJ, Faden RR, Nolan MT, Bodensiek E, Sugarman J. Medical and Nursing Students’ Television Viewing Habits: Potential Implications for Bioethics. Am. J. Bioeth. 2008;8:1–8. | To determine the television viewing habits and impressions of bioethical issues in televised medical dramas among medical and nursing students. This will help to determine the possible utility of medical dramas for bioethical learning, but this study does not aim to establish the actual effects of televised medical dramas on the bioethics-related attitudes and practices of medical and nursing students. | Web-based survey designed to gather data on basic demographic characteristics, general television viewing habits, television medical drama viewing habits, impressions of bioethical and professionalism issues in television medical dramas, sources of education about bioethics, and sources of information on bioethical issues. Medical & nursing students completing demographics (n=374), with a portion continuing to rest of survey (n=248, 29% response rate). | Research  Medical & nursing students | Frequency of medical drama viewing; which shows out of 4 specific programs (ER; Grey’s Anatomy; House, M.D.; and Nip/Tuck); compared recall of ethical issues discussed in medical dramas between pre-clinical and clinical medical/nursing students; and sources of information on bioethical issues. | More than 80% of medical and nursing students watch television medical dramas. The extent of clinical experience influenced students' perception of the adequacy of handling of ethical issues as portrayed on TV shows (students with more clinical experience tended to have more negative compared to students without clinical experience). More than 50% of viewers watch with friends and approximately 50% discussed these ethical issues with friends, which supports the contention that medical dramas may be an important part of “the informal curriculum.” Students almost universally rated television medical dramas as an insignificant source of information about bioethics. Authors suggest future study evaluating students' impressions of bioethical issues before/after medical drama viewing to more precisely measure impact of programs on beliefs & attitudes. | 1 |
| Czarny MJ, Faden RR, Nolan MT, Bodensiek E, Sugarman J. Response to open peer commentaries on “Medical and nursing students’ television viewing habits: potential implications for bioethics.” Am. J. Bioeth. AJOB. 2008;8:W1. | Czarny et al's response to and Ward & Sommers' and Trachtman's commentary that television depictions of bioethical issues are poor tools to be used in the teaching of bioethics to medical and nursing students. The authors are pleased their article has encouraged meaningful discussion about the potential role of televised medical dramas in ethics education for medical & nursing students. The authors underscore that their survey was designed to better understand the viewing habits of medical & nursing students and not to offer data to inform this important and related discussion. | N/A (not a study) | Perspective  Follow-up commentary/discussion on the topic for educators & learners. | N/A | N/A | N/A |
| Czarny MJ, Faden RR, Sugarman J. Bioethics and professionalism in popular television medical dramas. J. Med. Ethics. 2010;36:203–6. | To assess the possible influence of televised medical dramas on the perceptions of the general public and their potential utility in the education of medical and nursing students through content analysis of what is portrayed in these television programmes. | Constructed a list of all medical dramas being broadcast in the USA from the autumn 2005 to spring 2006 television season on broadcast or major cable stations, including BET, Univision and Telemundo, and that were available on DVD. House (season 2; 24 episodes),19 Grey's Anatomy (season 2; 26 episodes),20 ER (season 12, 22 episodes)21 and Nip/Tuck (season 3; 15 episodes)22 fit these criteria. ER was excluded because it was only available on DVD through the 2001–2002 television season.  Conducted a content analysis of House and Grey's Anatomy, focusing on incidents in the programmes that involved ethical issues ('bioethics') and questions of interpersonal relations ('professionalism'). | Research (qualitative review of TV medical dramas with content analysis) | Did not measure outcomes, as no study population was involved. This was a review of televised medical dramas with content analysis to understand the content available on topics of bioethics and professionalism. | Content analysis of Grey's Anatomy and House revealed that television medical dramas are rife with depictions of bioethical issues (179 distinct depictions of incidents) and egregious deviations from the norms of professionalism (327 incidents). They contain exemplary depictions of professionalism to a much lesser degree (69 incidents). TV medical dramas could help to engage students in discussions of the ethical handling of such issues when confronted in the practice of medicine. Future scholarship should be directed at assessing the effect of bioethics and professionalism in medical dramas on the attitudes and behaviour of patients and healthcare professionals as well as the role that these programmes could play in the education of future healthcare professionals. | N/A |
| Darbyshire D, Baker P. A systematic review and thematic analysis of cinema in medical education. Med. Humanit. 2012;38:28–33. | A systematic review of the current literature to see what is known within the published literature about the use of cinema in medical education with thematic analysis to determine topics investigated and gaps for future study. | Systematic review of six databases was carried out (AMED, BNI, CINAHL, EMBASE, MEDLINE from PubMed and PsycINFO) in November 2009. Keywords used were: cinema, film, movies, Hollywood, cinemeducation and medical education. All article types, from reports of randomised controlled trials through to letters, were examined. No date exclusion was imposed. | Research  Suitable systematic review article for educators & learners. | 20 articles included for review. The authors summarize which topic areas that have been covered, where studies are being conducted, target population, and specific movies used. | A number of studies have been conducted in this area, but more descriptive accounts of pedagogy needed. Authors encourage more educators to share their experiences to build a sound evidence base for the development of movies in medical education. | N/A |
| Datta V. Madness and the movies: an undergraduate module for medical students. Int. Rev. Psychiatry. 2009;21:261–6. | To understand the use of film as an educational tool in psychiatry and developing cultural sensitivity, and to critically examine how the social, cultural, and historical context of a movie impacts on the representation of mental illness, psychiatry, and psychiatrists. | 10 week special study module; weekly theme with film(s) focused on that theme. Students to do relevant readings & view at least 1 film (list of weekly films & course programme example provided). 2 hr seminar, with viewer-response criticisms and comparing/contrasting representation of mental illness, psychiatry & psychiatrists in the film(s). Small sample completed the course (n=8), with evaluation of the module through 19 item questionnaire; 14 Likert scale (1-6 strongly disagree/agree) and 5 qualitative questions. | Research  3rd year medical students | Student satisfaction with the course and knowledge of select psychiatric topics. | Students perceived the module as enjoyable and would recommend to other students. They self-assessed increased knowledge of psychiatry in historical context and psychiatric topics (better understanding of core curriculum topics, eg. schizophrenia, controversies in psychiatry, understanding of portrayal of mental illness/psychiatry/psychiatrists). This study provides provisional support that movies are a powerful educational tool in undergraduate psychiatry, especially for highlighting the significance of the social, cultural and historical context in which people become mentally ill and are treated, or are not treated as the case may be. Further research with objective assessment of knowledge needed. | 1, 2 |
| Dave S, Tandon K. Cinemeducation in psychiatry. Adv. Psychiatr. Treat. 2011;17:301–8. | Authors provide a detailed and useful on outline how and why educators should use cinema to teach psychiatry. Authors provide lists of films that correspond to specific mental illnesses. Authors posit that movies provide multiple perspectives and are useful to teach sensitive clinical issues (eg. countertransference, risk assessment) in a safe and ethically uncomplicated environment. | N/A (not a study) | Perspective  Guide for educators, but suitable for learners | N/A | N/A | N/A |
| Desai MS, Desai SP. Alternate methods to teach history of anesthesia. Anesth. Analg. 2014;118:438–47. | To describe the various methods that can be used to teach the history of anesthesiology (besides lectures), elaborating on books, movies and other non-lecture based modalities. | Brief description of non-lecture based methods includes movies *(The Great Moment*), TV series ("You are there"), and documentary (*Yankee Dodge, Ether Monument*), as well as historical narratives, novels, and museum/historic site tours. | Description  Anesthesiology residents | No outcomes assessed or reported. | The article describes alternate modalities of teaching the history of anesthesiology for educators to consider. The authors posit that these informal modalities can be implemented without much effort or take away from traditional curriculum. The authors are sharing their experiences and resources, but do not describe in detail outcome or evaluation of the use of novels, movies, documentary, and tours. | N/A |
| Dobson R. Can medical students learn empathy at the movies? BMJ. 2004;329:1363. | Summary of Glenn Flores' article "Doctors in the Movies," with list of top 10 movies most useful for medical education and best portrayal of doctors. | N/A | Perspective Commentary for medical and health professional educators. | N/A | N/A | N/A |
| Elder NC, Schwarzer A. Using the cinema to understand the family of the alcoholic. Fam. Med. 2002;34:426–7. | To describe the use film clips to teach about (1) alcoholics' impact on family members, (2) roles/problems that family members adopt, and (3) how literature/arts can lead to better understanding of alcoholism. | Brief description of the 45-90 minute course using film clips to teach about families with an alcoholic member. Format: group review of topic, group video viewing, and group discussion. Topics: role of the enabler, denial, isolation, project, family rules, family roles, (eg. hero, scapegoat), emotional experiences of the alcoholic family (anger, fear, betrayal, responsibility), and physical/emotional illnesses that may be associated with the alcoholic family (addiction, obesity, depression, anxiety, stress, marital discord). | Description  Family Medicine clerkship; residents, practicing physicians, and other health professional | Discussion topics that arose are described, but no formal evaluation reported. | No evaluative data. Description states that learners see film clips as an "interesting way to achieve understanding of the topic," including how to identify, acknowledge, and offer emotional support when they suspect family alcohol problems, community resources, skills of healthy detachment, and "do's and don'ts" for the alcoholic family. | 1 |
| Farré M, Arribas S, Perez J, Banos JE. Bioethical principles, clinical research and popular movies. Med. Educ. 2013;47:1141–2. | To describe the use of movies to introduce bioethical principles in human research to undergraduates. | Movie viewing (*Miss Evers' Boys, Extreme Measures*) in small groups followed by 1 hour discussion facilitated by tutor with topics on autonomy, justice, beneficence and non-maleficence. | Description  Undergrad students | No outcomes assessed or reported. | No description of how they evaluated/assessed outcome. Authors note both movies useful for bioethical principles, though not all euqally understood (infringement of the beneficence principle was less recognised). Authors share how educators should use movies to teach bioethics: (1) careful choice of movie,(2) prepare list of questions to enhance participation, and (3) evaluation of short essays may help assure learning objectives achieved (not clear whether authors evaluated essays for student knowledge). | N/A |
| Farré M, Bosch F, Roset PN, Baños J-E. Putting clinical pharmacology in context: the use of popular movies. J. Clin. Pharmacol. 2004;44:30–6. | Authors make a case for the benefits of using popular movies to illustrate psychological and sociological conflicts of medical practice. Provides detailed example of teaching objectives & questionnaire when using movies to teach clinical pharmacology. Uses 3 movies as examples: (*Lorenzo's Oil, Awakenings,* and *Miss Evers' Boys*. | N/A (not a study) | Perspective  Guide for educators, but suitable for learners | Student knowledge of pharmacology. | No actual study or data. Authors posit popular movies may increase students' motivation and desire to learn, and is therefore valuable as a teaching tool in pharmacology. | N/A |
| Flores G. Mad scientists, compassionate healers, and greedy egotists: The portrayal of physicians in the movies. J. Natl. Med. Assoc. 2002;94:635–58. | To analyze cinematic depictions of physicians to determine common demographic attributes of movie physicians, major themes, and whether portrayals have changed over time. | All movies released on videotape with physicians as main characters and readily available to the public were viewed in their entirety. Initial list compiled of movies with physician as protagonist by using VideoHound's Golden Movie Retriever 1999, with items under categories: Doctors and Nurses; Hospitals and Medicine; Shrinks; AIDS; and Evil Doctors. 505 movies were identified. Excluded movies were children's movies, X-rated movies, pilots of television shows, and movies whose main characters were: dentists, psychologists, nurses, PhD academics without MD. If the physician was not the protagonist, the movie was also excluded. 131 movies were viewed in entirety. Physician characteristics, diagnoses, and medical accuracy, with dialogue concerning physicians was transcribed. | Research  Detailed review of movies (guide) for educators, but suitable for learners. | Information abstracted for each of the 131 movies viewed included: genre (drama, horror, western, etc); descriptive adjectives about physician portrayal (eg. healer, idealist, mad scientist, greedy egotist); physician specialty, gender, ethnicity, age; country of origin of movie; medical setting; diseases discussed in movie; medical accuracy; and industry awards. | Physician movies were most often categorized as dramas (48%; n=63), comedies (21%; n=28), or horror films (14%; n=18). The typical movie physician is a White male surgeon in his 30's practicing at a hospital. Physicians were more likely to be male (p < 0.00001) and White (p < 0.00001). Only 15% were female and only 9% of all movie physicians were of non-White ethnicity. More than three-quarters of film physicians are surgeons, psychiatrists, or family/general practitioners. About half of all film physicians were portrayed in a positive light. The descriptive adjective "compassionate healer" applied to 56% of cinematic physicians. The other 44% of film physicians were portrayed negatively. The "mad scientist" was the most common negative depiction (20%). Medical inaccuracies were noted in 27% of physician films, and fell into three categories: (1) unrealistic depictions or views of physicians or their practice; (2) scientifically unsubstantiated therapeutic interventions; and (3) factual errors. Portrayals of physicians as egotistical and materialistic have increased, whereas sexism and racism have waned. Critical issues explored were medical ethics and managed care. Authors conclude that negative cinematic portrayals of physicians are on the rise, which may adversely affect patient expectations and the patient-physician relationship. Nevertheless, movies about physicians can serve as useful gauges of public opinion about the medical profession and as tools for medical education. | N/A |
| Flores G. Doctors in the movies. Arch. Dis. Child. 2004;89:1084–8. | To review how doctors are portrayed in movies and the 10 most useful films for medical education. | An update on author's 2002 in-depth analysis of the portrayal of doctors in movies. | Research  Review (guide) for educators, but suitable for learners. | N/A | Author describes movies to demonstrate each identified theme, including: money and materialism, paediatricians, and bureaucracy and healthcare systems. Author provides an annotated top 10 list of movies with doctors that are "best" movies (*Red Beard)*, most humorous (M*A*S*H), and most useful for medical education (*The Doctor).* | N/A |
| Fritz GK, Poe RO. The role of a cinema seminar in psychiatric education. Am. J. Psychiatry. 1979;136:207–10. | To assess the value of commercial film as a useful medium in a psychiatric residency curriculum. | Elective, monthly film seminar. Includes spouses/friends to help balance traditionally male perspective in psychiatry. Questionnaire mailed to 36 residents. | Description  Psychiatry residents | Questionnaire on the value of the seminar. | Participants enjoyed the experience and felt that the discussion of the films broadened their perspectives to include humanistic, social and philosophical levels; related psychiatric insights to everyday life; useful opportunity for socialization with professional peers; increased knowledge of unconscious dynamics; stimulated creative thinking. | 1, 2 |
| Furst BA. Bowlby goes to the movies: Film as a teaching tool for issues of bereavement, mourning, and grief in medical education. Acad. Psychiatry. 2007;31:407–10. | Author presents an overview of normal and pathological mourning, emphasizing the work of John Bowlby. Author discusses movies that illustrate Bowlby's concepts and strategies on using these movies to teach students about bereavement and death through film (*Under the Sand, Ordinary People, Love Liza, The Son's Room)*. | N/A (not a study). Author mentions he conducted seminar where movies are screened for trainees and faculty followed by discussion to clarify psychological processes portrayed and link to clinical case material, as well as using movie clips to accompany teaching points in lecture on mourning. No assessment or formal evaluation reported. | Perspective  Guide for educators, but suitable for learners | N/A | N/A | N/A |
| Gallagher P, Wilson N, Jaine R. The efficient use of movies in a crowded curriculum. Clin. Teach. 2014;11:88–93. | To teach key public health issues to medcial students through movies and demonstrate that when movies are used innovatively, they enhance student understanding of medicine with minimal impact on a crowded curriculum. | During a 5 week long Public Health module, students borrow movies with a public health message. During the last week, a 1.5 hr session is allocated for students to give a brief synopsis of the movie and highlight the key public health issues dramatized in the movie. Surveys were completed (n=78 out of 84 students in the year group) and field notes made during each group discussion. | Research  4th year medical students | Student reaction to the course (how many movies watched, if more than one, reasons for not watching more than one, comparing movie watching to novel reading) and student knowledge (public health issues, impact of movies [whether they thought about the movie/issues], and whether movies help medical students learn about health and social issues). | Nearly 54% watched more than one movie when obligated to watch only one and usually discussed public health and social issues of the movie with other people (54% for the first movie selected). When asked if watching movies helped medical students learn more about health and social issues, most ‘agreed’ (55%) and a third ‘strongly agreed’ (33%). Qualitative comments from students were that the intervention was an enjoyable way to learn, made them reflect on various issues and make links to other course material, and improved general knowledge on public health. Authors found that the use of commercial movies appeared to encourage both independent and collaborative learning, promoted discussion, integrated subjects and was efficient in the use of scheduled class time. | 1, 2 |
| Glasser B, Clark M, Greenhalgh T, Harmar-Brown C, Leach J, Modell M, et al. From Kafka to Casualty: Doctors and medicine in popular culture and the arts - A special studies module. J. Med. Ethics. 2001;27:99–101. | To describe a medical humanities module to promote student understanding of professional identity and develop analytic/interpretive skills by appreciating range and disparity between professional self-image and popular conceptions of doctors/medicine. | 2-week special studies module, small-group work and guided private study. One session is on "Doctors in the movies" which uses multiple movie clips and another session on "Doctors on the box: Casualty" where medical drama *Casualty* is used to discuss authenticity. Readings or movie/television clips are accompanied by discussions. Formative assignments like creative writing or review of literature/movie encountered in the course assigned. | Description  3rd year medical students | No formal assessment conducted. Tutor feedback about student effort/achievement was phoned in each day to the course organizer. | No formal evaluation, but authors state that "the course has received consistently positive evaluation" and that tutors enjoy teaching it (no one has exercised their annual right of opting out of contributing to the course). The medical school has discussed more frequent offerings of the course. Authors note the course's success relies on traditional pillars of planning and teaching skills, not only on its innovative content. | 1 |
| Goldman JD. An elective seminar to teach first-year students the social and medical aspects of AIDS. Acad. Med. 1987;62:557–61. | To acquire accurate information about AIDS, to overcome fear of dealing with AIDS patients and discomfort in dealing with homosexual men, and to learn about/feel empathy for persons with AIDS. | Elective, 7-week seminar (12 hours) using documentary films, readings, guest speakers with AIDS, and roundtable discussion as part of comprehensive teaching program. | Description  1st year medical students | Questionnaire with 11 Likert-type (5-point scale) questions and 1 open-ended question. | The students have reported the seminar to be valuable in helping them overcome their fear of the disease, develop empathy for patients with catastrophic diseases, understand a comprehensive approach to a complex disease, and transfer empathy to patients with other catastrophic illnesses. | 1, 2 |
| Gorring H, Loy J, Spring H. Cinemeducation: Using film as an educational tool in mental health services. Heal. Inf. Libr. J. 2014;31:84–8. | To describe cinemeducation (movies in medical education) to teach about mental health. Focus on how the particular mental health condition or treatment was portrayed, and how mental illness is used in film for plot development, narrative and dramatic conflict. Movies included *Requiem for a Dream, Iris, We Need To Talk About Kevin, Shock Corridor, Regeneration, Birdy, Away From Her* and *Transamerica.* | Brief description of 2 separate cases of movie clubs. Film viewing followed by discussion. The first case had reflection sheets that counted as additional activity in psychiatry placement log books. | Description  1st case: Medical students, staff  2nd case: Medical students, staff, friends, family | Qualitative responses from students are presented, though unclear how this data was collected (no description of whether/how evaluation was done). | No description of how outcome/evaluation was assessed. Authors include some movie club attendee quotes, enthusing that they learned about Asperger's (*Away From Her*), were able to observe uncommon textbook symptoms and able to understand stigma of mental health (*Shock Corridor*). | 1, 2 |
| Graf H, Abler B, Weydt P, Kammer T, Plener PL. Development, implementation, and evaluation of a movie-based curriculum to teach psychopathology. Teach. Learn. Med. 2014;26:86–9. | To assess the effect of a movie seminar on students’ attitudes toward psychiatry and to measure the effectiveness of knowledge transfer. | Elective seminar with introduction on psychiatry in movies and ICD-10. 9 weekly lessons of 90 mins focused on subchapter of psychiatric diagnostic entities (Chapter "F") of the ICD-10. Students viewed movie clips, discussed psychopathological symptoms based on current knowledge, and lecturer provided additional information on psychopathological symptoms as described in ICD-10. Students were encouraged to identify described symptoms while viewing more movie clips, followed by discussion on movie portrayal versus real life presentations that psychiatrist/neurologist lecturers provided to help students reflect on their attitudes of psychiatry from movies versus lecturers' experiences. teach med students psychopathology/ psychiatry. Written test on ICD-10 criteria given at the end of the semester to test student knowledge. Used pre/post-video Attitudes towards Psychiatry (ATP35) scale to determine changes in attitudes toward psychiatry and knowledge gains (n=54 students volunteered to take part in pre/post-testing out of 75 seminar participants) | Research  Medical students | Student attitudes towards psychiatry and the seminar, and student knowledge about ICD-10 criteria. | As expected for an elective seminar, attitudes toward psychiatry were already clearly positive at pretesting. However, a further significant improvement (p = .012) was observed in the posttest. In the pretest of student’s knowledge of ICD-10 criteria, students achieved a mean of 15.7 (SD=3.402), 95% CI [14.78, 16.63] of a maximum of 25 possible points. Post-seminar, they achieved a mean score of 22.1 points (SD=2.304), 95% CI [21.48, 22.74], resulting in a significant increase of knowledge (df = 53, p < .001). Feedback forms returned with positive feedback on the atmosphere of the seminar, the enthusiasm of lecturers, and the learning material that was provided online. Criticisms included discussing multiple diagnoses of one F-category within a session and the use of more than one film per topic. Authors conclude that teaching psychopathology through the use of movies can improve attitudes toward psychiatry as supported by overall positive changes in the ATP-35 (ATP = Attitudes Towards Psychiatry scale) and increase knowledge about diagnostic criteria in psychiatry. | 1, 2 |
| Gramaglia C, Jona A, Imperatori F, Torre E, Zeppegno P. Cinema in the training of psychiatry residents: focus on helping relationships. BMC Med. Educ. 2013;13:90. | To describe a new way to teach with movies using a symbolic and allegorical approach, based on Jung and Hillman’s considerations about the relation between images and archetypes, and archetypal experience and learning. Instead of focusing on a variety of psychiatric disorders shown through film, movies can develop learners' awareness of own emotions and empathy, a fundamental determinant of quality in medical care. | Description of Jung and Hillman's theory, the theoretical basis of this course, Includes list of movies used and description of the themes/discussion points for each movie (issues from theoretical premises including: meaning of illness, good/bad helping relationships, prejudice, anima and feeling). Ongoing cinemeducation seminars every 2 weeks (n=70 medical students, n=12 psychiatry trainees). | Description  Psychiatry residents and Medical students | Structured assessment underway; no formal outcome/evaluation yet. Authors report their impressions. | No formal evaluation data, but authors report their impressions that participants were enthusiastic and highly involved with these sessions, as evidenced by their enthusiasm in discussion. Authors state that in their experience, teaching with movies effectively increased students' sensitivity and raised their awareness that they need education in order to improve their relational skills and empathy. This teaching method helped participants reflect on deeper and broader questions about the meaning of being a health-care provider and develop psychiatrists who relate to patients and families empathically and compassionately. | N/A |
| Greenberg HR. Caveat actor, Caveat emptor: some notes on some hazards of Tinseltown teaching. Int. Rev. Psychiatry. 2009;21:241–4. | Author articulates reasons why movies should not be used to teach psychiatry. Author provides detailed examples of misrepresentation and inaccuracies in movies of psychiatry and psychotherapy. | N/A (not a study) | Perspective  Article aimed at educators, but may be of interest for learners. | N/A | N/A | N/A |
| Guerrero APS, Jamora MJ. The fall and redemption of people and systems: Potential lessons from the “Star Wars” saga. Acad. Psychiatry. 2007;31:485–90. | This article discusses how Star Wars movie clips can be used to teach medical students or psychiatry residents about resilience, leadership, administration, liaison, identifying certain psychiatric traits, identifying child/adolescent psychiatric disorders, health care quality, medical error, and clinician teamwork. | N/A (not a study) | Perspective  Suitable guide for educators & learners. | N/A | No study data provided, although authors note their own use of Star Wars so far has been brief video clips to stimulate discussion. No outcome/evaluation measured. | N/A |
| Halamek LP. Lost moon, saved lives: Using the movie Apollo 13 as a video primer in behavioral skills for simulation trainees and instructors. Simul. Healthc. 2010;5:303–10. | This article champions the use of video to teach behavioral skills. Halamek develops teaching points & debriefing for 3 scenes from the movie *Apollo 13* as an example of how popular movie clips can be used for facilitated group or independent learning, providing context and key points for discussion. | N/A (not a study) | Perspective  Suitable guide for educators & learners. | N/A | N/A | N/A |
| Hallberg J. 8 films medical students should see. Minn. Med. 2007;90:28–32. | Author briefly describes the power of movies to engage viewers completely and teach about justice, ethics, yearning, love, and racism beyond traditional curriculum. Author asks fellow faculty to recommend 8 movies they would recommend to medical students about what it means to be a physician. | N/A (not a study) | Perspective  Guide for medical students, but suitable for educators as well. | N/A | N/A | N/A |
| Henry M, Newman J. Movies and the medical profession. Clin. Teach. 2009;6:269–71. | To describe the use movies and documentaries to generate discussion and learn about various topics in medicine, including professionalism, alcoholism, public health, mental illness, anatomy, racism, and medical experimentation. | Evening movie series. Format was brief introduction, movie screening, and facilitated discussion. Average attendance of 60 people, with a range of 38–92 attendees. | Description  Medical students and other disciplines | No formal evaluation or assessment conducted. | Authors provide a guide using 3 movie examples and discussion points for readers interested in using this method of teaching. Authors conclude that movies were very effective at generating discussion and encouraging learning, but no objective evaluation is provided. | N/A |
| Hirt C, Wong K, Erichsen S, White JS. Medical dramas on television: a brief guide for educators. Med. Teach. 2013;35:237–42. | To systematically review episodes from medical dramas and provide a guide to how each drama is best suited for particular educational use (eg. *ER* and *Scrubs* for teaching & learning; *House* and *Grey's Anatomy* for teamwork & ethics), with the goal of encouraging integration of this material into medical education. | 177 episodes from 8 popular television medical dramas produced between 1990-2009 were systematically viewed and analyzed for material suitable for integration into medical education. Medical dramas viewed: *Northern Exposure, Cardiac Arrest, ER, Scrubs, House, Doc Martin, Grey's Anatomy,* and *Nurse Jackie*. | Perspective  Guide for educators, but suitable for learners | N/A | This is not a study measuring student perceptions or knowledge, and there is no description of evaluation of any course based on the use of medical dramas set out in the guide. Authors note that medical dramas hold much promise for engaging learners in a new way by providing compelling story-telling in a variety of medical contexts. This guide provides a useful description of each television show, with suggestion of how each drama's topics can be used in medical education. | N/A |
| Hojat M, Axelrod D, Spandorfer J, Mangione S. Enhancing and sustaining empathy in medical students. Med. Teach. 2013;35:996–1001. | To test the hypotheses that medical students’ empathy can be enhanced and sustained by targeted activities. | 2-phase study with medical students (n=248) **Phase 1**: Experimental group watched and discussed video clips of patient encounters meant to enhance empathic understanding. Control group watched a documentary film. **Phase 2 (10 weeks later): E**xperimental group students divided into two groups. One group attended a lecture on empathy in patient care, and the other plus the control group watched a movie about racism. The Jefferson Scale of Empathy (JSE) was administered pre-post in Phase 1 and posttest in Phase 2. | Research  Medical students | Student empathy scores. | Findings of the present study are encouraging in showing that it is possible to enhance and sustain empathy in medical students by targeted educational programs. **Phase 1**: average increase of 2.2 scale points was observed in the empathy scores of students in the experimental group, which was statistically significant (p50.01) (effect size¼0.21). No change in mean empathy score was noticed in the control group.  **Phase 2:** experimental group who attended the lecturer session on empathy, thus, being exposed to supplementary empathy-enhancing reinforcement (reinforced group), the statistically significant improvement in empathy scores (from pretest to posttest 1) was sustained in posttest 2 ( pretest < posttest 1=posttest 2, p<0.01). No statistically significant change in empathy scores was found in the control group (pretest=posttest1 = posttest 2). | 2 |
| Hyler SE, Schanzer B. Using commercially available films to teach about borderline personality disorder. Bella Bull. Menn. Clin. 1997;61:458–68. | Authors describe 33 films that depict different aspects of borderline personality disorder and suggest the films' uses for illustrating DSM-IV diagnostic criteria. | N/A (not a study) | Perspective  Suitable guide for educators & learners. | N/A | N/A | N/A |
| Johnson JM, Beresin E V., Stern TA. Using Breaking Bad to teach about defense mechanisms. Acad. Psychiatry. 2014;38:716–9. | To use episodes of Breaking Bad to teach psychiatry residents about different kinds of defence mechanisms. | Format: Lecture reviewing basic theory of defence mechanisms, discussing plot details from *Breaking Bad,* screening scenes as detailed in the article, followed by trainees identifying and commenting on defence mechanisms. | Description  Psychiatry residents (PGY-3) | No formal evaluation or assessment conducted. Authors gathered informal feedback from students. | Authors state that residents reported satisfaction in learning about defence mechanisms, particularly using a popular television show. Residents reported improvement in their ability to characterize and understand defences within their own individual cases of psychodynamic psycho-therapy. Authors suggest future efforts to incorporate pre/post surveys to document objective effectiveness of this format in teaching about defence mechanisms. | 1 |
| Jukić V, Brečić P, Savić A. Movies in education of psychiatry residents. Psychiatr. Danub. 2010;22:304–7. | To describe how movies can be used to teach historical and current representations, as well as issues and possibilities related to psychiatry, mental illnesses, and doctor-patient relationships. | Educational forum "Movies and Psychiatry" comprising of movie viewing followed by semi-structured discussion moderated by invited commentators, movie critic, and psychiatrist. 1st session *One Flew Over the Cuckoo's Nest* (n=130), 2nd session *A Beautiful Mind* (n=50), and 3rd session *Mr. Jones* (approximate n=50). | Description  Psychiatry residents, psychiatrists psychologist, students | No outcome/evaluation clearly assessed or described. Authors relate their impressions and observations of the first three "Movies and Psychiatry" sessions. | Authors observed very active participation in discussion by participants, with residents exposed to various clinical representations schizophrenia *(A Beautiful Mind*) and bipolar disorder *(Mr. Jones),* and discussions about past and present problems of psychiatric services and treatments (*One Flew Over the Cuckoo's Nest*). Psychiatry residents show creativity and great interest by their numerous proposals for future panel discussion. The authors state that psychiatry residents accept this form of teaching and movies can be used to teach principles of doctor-patient interaction and psychopathology, to understand context in which psychiatry is functioning within society and other aspects of psychiatry practice. | 1 |
| Kalra G. Psychiatry movie club: A novel way to teach psychiatry. Indian J. Psychiatry. 2011;53:258. | To describe how movies can be used as a tool for training psychiatry residents with movies as case presentations of psychopathology. Includes list of movies that can be used to teach schizophrenia, delusional disorder, mood disorder, anxiety disorder, personality disorder, and substance use disorder. | Elective psychiatry movie club. Small group would view movie with psychiatric relevance, followed by a detailed discussion about positive points and criticisms of psychiatric illness, patient, and mental health professional portrayed in the movie. | Description  Psychiatry residents | No formal evaluation or assessment conducted | Author states drawback of this method is that movies are made for entertainment, not education, and can therefore represent how individual writers/directors conceive of mental illness rather than presenting scientific understanding. Author states that the trainees welcomed movies as a teaching tool and the movie club continues. | N/A |
| Kalra G. Teaching diagnostic approach to a patient through cinema. Epilepsy Behav. 2011;22:571–3. | Description about how the movie *Stigmata* can be used to teach students about diagnostic dilemmas and approach to patients presenting with difficult clinical syndromes. | Describes the movie events in relation to diagnostic approaches to: nonepileptic attack disorder, malingering, complex partial seizures with Gastaut-Geshwind syndrome, schizophrenia, psychosis not otherwise specified, and psychogenic purpura. | Perspective  Suitable guide for educators & learners. | N/A | N/A | N/A |
| Kalra G. Talking about stigma towards mental health professionals with psychiatry trainees: A movie club approach. Asian J. Psychiatr. 2012;5:266–8. | To help psychiatry trainees talk about their experiences with stigma towards mental illness and their profession. The author encourages readers to explore these issues through similar movie clubs in their departments. | Elective psychiatry movie club with voluntary trainee participants (n=11). General discussion about medical practitioners with psychiatric disorders, questions to guide trainees in discussing the issue of stigma towards psychiatry as a specialty, movie screening of *Gothika* with pauses at specific clips, further discussion. | Description  Psychiatry residents | No outcomes measured directly. Author includes some trainee responses to the guided questions asked pre-movie screening that reflect their personal experiences of stigma towards their specialization, but did not gather data to evaluate reactions to the effectiveness of the movie club. | No outcome/evaluation measured. Author provides trainee responses to pre-movie screening questions that affirm stigma towards psychiatry specialization (eg. all trainees answered "yes" to the question "When you expressed interest in psychiatry, you were seen as odd and eccentric.") Author includes some trainee quotes from post-movie screening discussion that reflects their experience of stigma attached to psychiatry. Author concludes the psychiatry movie club encouraged the psychiatry trainees to be honest and share their experiences by speaking out how they felt. | N/A |
| Kaye DL, Ets-Hokin E. The Breakfast Club: Utilizing popular film to teach adolescent development. Acad. Psychiatry. 2000;24:110–6. | To describe teaching psychiatry residents about adolescent development through movies, using *The Breakfast Club* as an example. | Description of "The Breakfast Club" seminar, where sometimes movie is shown in entirety, followed by subsequent teaching sessions that look at specific scenes. Other times, movie is shown, pausing to discuss relevant teaching issues as they occur. Discussion is accompanied by supplementary readings for in-depth theoretical background. Authors provide film synopsis and detailed discussion points and developmental themes raised in scenes from the movie. | Description  Psychiatry residents | No detail on how (if any) formal evaluation was done. | Authors state that residents and faculty have enjoyed this approach to teaching and learning. No formal student satisfaction or knowledge assessed or reported. | N/A |
| Klemenc-Ketis Z, Kersnik J. Using movies to teach professionalism to medical students. BMC Med. Educ. 2011;11:60. | To test the relevance and usefulness of movies in teaching professionalism to 4th year medical students and to assess the impact of this teaching method on students’ attitudes towards select professionalism topics. | A 4 month elective course with topic of "professionalism in medicine." 11 students participated, with 8 female students (66.7% of the group). The mean age of the students was 21.9 ± 0.9 years. Method was lecture, group work, plenary discussion with home movie viewing. Students’ written essays and oral presentations formed the basis for qualitative analysis using thematic codes. | Research  4th year medical students | Outcome measures were student satisfaction (reflected in attendance) and film specific professional themes in discussion with elaboration in written essays. | Students recognized the following professionalism dimensions in movies: importance of doctor patient communication, empathy as a mile stone of doctor patient relationship, doctors' selfish personal interests and importance of palliative care. The reported dimensions correspond to the main components of medical professionalism, which are excellence, humanism, accountability and altruism. Movies also engaged students in thinking about their attitudes towards life, death and phases of dying. Authors concluded that movies help students have safe space to explore their values, beliefs, and attitudes toward features of professionalism. | 1, 2 |
| Koren G. Awakenings: using a popular movie to teach clinical pharmacology. Clin. Pharmacol. Ther. 1993;53:3–5. | To describe a program where the movie *Awakenings* was used to teach medical students, science undergrad and science graduate students about clinical pharmacology. | Authors provide 10 detailed questions for educators to help students address concepts of pharmacokinetics, pharmacodynamics, adverse effects, ethics of research, and expectations of patients through viewing *Awakenings*. Authors used this method to teach clinical pharmacology to 1st and 2nd, but also postgraduate students. | Description  Medical students | Nothing measured directly, only a description of program. The program has been tested for feasibility, general acceptance, and satisfaction, but no formal surveys. | No outcome/evaluation measured, though authors state "this program has achieved high levels of satisfaction among medical students and graduate and postgraduate trainees in clinical pharmacology." The detailed description of using a movie to teach clinical pharmacology with detailed, focused questions is an example of incorporating a historic case through a movie into the education process. | N/A |
| Kuhnigk O, Schreiner J, Reimer J, Emami R, Naber D, Harendza S. Cinemeducation in psychiatry: A seminar in undergraduate medical education combining a movie, lecture, and patient interview. Acad. Psychiatry. 2012;36:205–10. | To enable 3rd year medical students to gain a deeper understanding of psychiatric illness in order to dispel students' negative attitudes toward psychiatry. | A “cinemeducation seminar,” one of six mandatory modules chosen between 3rd-5th year of 6-year undergraduate medical curriculum. 4 hour seminar comprised 3 elements: 1) short introduction + movie viewing, 2) discussion about movie + short lecture about specific psychiatric disease seen in movie, focusing on diagnosis based on the ICD-10/DSM-IV and therapy, and 3) patient interview with student participation. Evaluation questionnaires from 12 trimesters (Jan2005-Dec2008), covering 39 seminars were included in this study. A total of 1,032 students (approximately 33% of all medical students in the average clinical-year group) returned the questionnaires. | Research  3rd year medical students | General satisfaction with this new teaching format, selection of the movies, the achievement of the learning objectives, the didactic quality of the teachers, and organizational matters were included. Participant’s agreement or disagreement with each statement was to be indicated on a 6-point Likert scale. | All of the calculated means were within the positive range (1–3) of the 6-point Likert scale, with students particularly valuing the combination of movie and seminar (1.4 [SD: 0.7]), and felt that the cinemeducation seminar was useful for giving them access to the subject of psychiatry (1.5 [SD: 0.7]). All learning objectives were ranked within a satisfactory range (1.8–3.0), with “I gained an impression of the treatment options for the specific disease” being given the lowest rating (3.0 [SD: 1.3]). The organization of the seminar as a whole was rated positively (1.5 [SD: 0.8]). From the students’ perspective, this type of seminar seems to be helpful for providing an impression of psychiatric illnesses and enabling students to put themselves in the position of a person suffering from these illnesses. Therefore, the authors judged the cinemeducation seminar to be an appropriate teaching format in undergraduate medical education. | 1, 2 |
| Lee Y-M, Ahn D-S. Medical-themed film and literature course for premedical students. Med. Teach. 2004;26:534–9. | To help students gain insight into their professional and personal lives as future physicians, including appreciating the humanistic and social aspects of medicine. | One three-hour session per week, over six weeks, for the entire class of second-grade premedical students (n=126). Instructional methods included lectures, small-group discussions related to books or films selected by the students, and presentations and panel discussions. Course evaluation for each class was conducted using feedback obtained after each session, and by completion of an end-of-course survey Students completed a short essay regarding the most impressive or valuable things learned from the class, and suggestions for improving the next session. | Research  Premedical students | (1) to broaden students’ perspectives on the life of physicians; (2) to understand medicine’s humanistic and social aspects; 3) to understand the illness experience from the patients’ perspective; (4) to give premedical students the opportunity to understand the relevance of literature to medicine; (5) to develop an awareness and understanding of medical issues as represented in literature and film; (6) to foster presentation and discussion skills; (7) to develop teamwork skills. | Students’ responses showed that this course helped them to gain perspectives on both a physician’s life and medical practice, and also enhanced their understanding of the humanistic and social aspects of medicine itself. Students reported that they also valued the advantages of collaborative learning, which holds the potential for improving their skills in presentation, discussion, communication and teamwork. Students reported that the films required less time and effort to study than the literature, but that literature provided a more in-depth commentary on the medical profession and more challenging dilemmas. This is most probably due to the fact that movies must have commercial dramatic appeal, and henceforth do not accurately reflect the medical profession in itself. | 1 |
| Lenahan P, Shapiro J. Facilitating the emotional education of medical students: Using literature and film in training about intimate partner violence. Fam. Med. 2005;37:543–5. | To educate medical students in identifying and intervening in intimate partner violence (IPV), while recognizing and coming to terms with students' own fears, anxieties, and negative judgments related to IPV scenarios. | Added 2 hour humanities-based session to standard IPV training. Medical students participated in a seminar where they were shown movie clips, read poems and asked to discuss and role-play specific situations afterwards. A survey or informal discussion (not specified in article) was held after where students evaluated the seminar and provided feedback. | Description  Family medicine clerkship | Student knowledge about identifying IPV, understanding IPV from the victims' and perpetrators' perspectives, and student enjoyment of the seminar. | Students became more knowledgeable about and capable of identifying IPV and intervention strategies, more comfortable with discussing it, and viewing IPV with greater empathy. Authors state "the seminar has been evaluated very positively by students" (no quotes or other data. | 1, 2 |
| Lepicard E, Fridman K. Medicine, cinema and culture: A workshop in medical humanities for clinical years. Med. Educ. 2003;37:1039–40. | To endorse a patient-centered view of medicine in students through a bio-psycho-social approach to disease and lifelong learning using full-length movies. | Description of a pilot program, "Medicine, Cinema and Culture," a workshop with 8 meetings with group viewing of an entire movie + 1 hour small group discussion. | Description  Clerkship students | Questionnaire with 3 questions: (1) To what extent did participation in the workshop offer an opportunity to discuss significant clinical experiences? (2) Did exposure to the different kinds of caregivers presented in the movies lead students to identify with a certain character? (3) To what extent did the workshop present different perspectives on the medical world? | No description of questionnaire results, but presumably rating of 3 questions related to student reactions of usefulness/relevance of program using movies. The authors state "positive results of the evaluation," leading to workshop being extended to all students in the following year. | 1 |
| Lim RF, Diamond RJ, Chang JB, Primm AB, Lu FG. Using non-feature films to teach diversity, cultural competence, and the DSM-IV-TR outline for cultural formulation. Acad. Psychiatry. 2008;32:291–8. | A literature review to describe the uses of documentary and instructional films to teach cultural competence, which is necessary in all 6 areas of ACGME competencies. Authors then share how they used these documentary and instructional films (*The Color of Fear, The Cross-Cultural Therapeutic Alliance, The Culture of Emotions*) to teach cultural competence. Includes list of films for cultural competence on issues of: race, gay/lesbian/bisexual/transgender, and diversity counseling. | Description of experience with best use of film through 2.5 hour session (30 mins eating, 90 mins film, 30 mins discussion). | Description  Literature review for documentary & instructional films, with brief description of authors' uses of the films in their teaching. | None directly measured, but authors describe students reactions to film: emotional reaction; use as a learning tool (awareness, sensitization); exemplar or role modeling; strategies for certain issues (eg. Eradicating racism); and raising awareness of privilege. | The authors state that documentaries and other non-fiction clips can be used to teach psychiatry residents about cultural competencies through diversity training. | N/A |
| Lim ECH, Seet RCS. In-house medical education: redefining tele-education. Teach. Learn. Med. 2008;20:193–5. | To discuss the educational merits of medical drama and describe a proposed module using episodes of *House* followed by discussion, suggesting that medical dramas can be effective in teaching professionalism and other "soft" skills. | Description of a proposed series of 1 hour lunchtime sessions called "In-*House* Medical Education Initiative.“ *House* will shown, followed by discussion of (de)merits of the show in relation to ethics, professionalism, history taking, clinical examination, and diagnostics (mis)steps. | Description of a proposed module for undergrad & postgrad students | Authors aim to launch the described module. No assessment yet as module has not yet been piloted. | Authors posit that that an entertaining and instructive show like *House* will attract students and focus learning on professionalism, ethics, and patient communication. Authors said would launch pilot project next year (2009) with assessments of efficacy and participant feedback, but no follow-up articles were found. | N/A |
| Loscos J, Baños J, Loscos F, de la Cámara J. Medicine, cinema and literature: A teaching experiment at the Universitat Autonoma de Barcelona. J. Med. Movies. 2006;2:138–42. | To use movies and literature as a teaching tool to help medical students understand patients in their entirety, "read and understand medicine" beyond the diagnostic level, with a broader appreciation of the emotional impact of disease. | Description of an elective course where movies and literature were used with facilitated discussion to provide students with medical situations that would develop a critical standpoint, stimulate emotional knowledge of illness, and knowledge of social/individual consequences of illness. | Description  3rd to 6th year medical students | Authors mention that students were assessed based on attendance, written papers, participation in discussion, and attitude during the course, but no data provided for the evaluation or assessment. | Authors quote student as saying that they were encouraged to have a course that focused on "patient treatment, patient-physician relationships and respect for our patients…which in the future we shall have to use routinely in our professional careers." Authors report a "high level of satisfaction of the students" with the course based on student attendance, essays, and participation in discussions (no data clearly described). Authors conclude that movies can help students reflect upon the humanitarian (emotional and psychological) aspects of medicine. | 1 |
| Lumlertgul N, Kijpaisalratana N, Pityaratstian N, Wangsaturaka D. Cinemeducation: A pilot student project using movies to help students learn medical professionalism. Med. Teach. 2009;31:e327–32. | To foster critical thinking skills, professionalism, and working cooperatively in medical students using cinemeducation (movies). | Cinemeducation Project organized, facilitated, and attended by students (n=20-30 students each session). Group met every other week to screen 4 movies (Patch Adams, Awakenings, Lorenzo's Oil, The Death of Mr. Lazarescu) and 2 episodes of *ER* with professionalism issues, followed by group discussion led by 2 students. Tailored questionnaire for each movie guided discussion. Assessment of the seminars through reflective discussion, with some participants writing opinions on an open-ended questionnaire. | Research  2nd year Medical students | Student knowledge, demonstrated through themes they identified and raised during reflective discussion (one of the evaluation processes - no clear description of any other evaluation processes though). Student organizer (staff) attitudes about involvement in the project also garnered through discussion. | No quantitative measures of satisfaction (for student organizers) or learning (student participants); results based on statements from reflective discussions. Participants stated that by watching scenarios from movies/medical drama *ER* and discussing issues, students developed their critical thinking and moral reasoning skills. In addition, they learned 5 main ethical issues in each film touching on professionalism (doctor–patient relationship, informed consent and clinical trials in patients, management of genetic disorders, patient management, and brain death and organ transplantation). Student organizers gathered after the last session to reflect on their overall attitude towards the project, including what they liked/disliked, what they had learned, and whether their attitudes had changed after completing this task. | 1, 2 |
| Mandel JB. Medical education about human sexuality: the impact of film in the workshop setting. Med. Educ. 1983;17:305–10. | To study the effectiveness of using "explicit films" in attitude change or knowledge increase about sexuality. | Pretest/posttest design with control group, randomly assigned, controlled for sex, to one of two 1-day human sexuality workshops (n = 19 Film/discussion, n =21 Discussion only) or a control group (n = 17 no workshop). Workshop topics: male and female psycho- sexual development, masturbation, variant lifestyles with emphasis on homosexual behaviour, and the role of touching. | Research  Medical students (n=65) | Analysis of pre- and post-workshop scores on Sexual Knowledge and Attitude Test (2nd Edition). | Post-test data from the attitude and knowledge sections of the SKAT test showed that participants in both the film and discussion and discussion-only workshops changed significantly more than the control group on the heterosexual relations (P< 0.05) and masturbation attitude scales (P< 0.01). No significant changes occurred on the knowledge section. Both groups felt that they had become more tolerant and more knowledgeable as a result of their experience and a majority of both groups felt that the workshops should be offered again. | 1, 2 |
| Fresnadillo Martínez JM, Amado CD, García Sánchez E, Sánchez García JE. Teaching methodology for the utilization of cinema in the teaching of medical microbiology and infectious diseases. J. Med. Movies. 2005;1:17–23. | To elucidate the methodology used in teaching microbiology to medical students using movies, with teaching design, objectives, suggested film selection criteria, methods of screening films, and evaluation. | Based on authors' experience of developing an elective course to teach medical microbiology, they describe a new approach: the study of a discipline (microbiology) through movies. Specific movies related to the topic are described, with learning objectives and lesson plan examples are given. | Description  Medical Students | No formal objective evaluation of student satisfaction or knowledge of microbiology (even though authors state they have used this methodology to teach this topic). | No study data reported. Authors state that the movies listed and the methodology described in detail works to teach microbiology through movies. | N/A |
| Mcneilly DP, Wengel SP. The “ER” seminar: Teaching psychotherapeutic techniques to medical students. Acad. Psychiatry. 2001;25:193–200. | To teach psychotherapy to medical students using *ER*, as a medical drama may reduce student resistance to learning psychotherapeutic techniques and so that students could apply/reinforce psychotherapeutic principles when watching future episodes of the program. | The *ER* seminar, "Psychotherapeutic Techniques I and II," took place in the 2nd week of a 6-week clerkship in psychiatry. Two 90 minute seminars. First part: Introduction to psychopsychodynamic, behavioral, and humanistic-existential theories, reminded of DSM-IV diagnostic criteria for personality disorders, with medical drama clips shown to aid facilitated discussion on "difficult" patients. 5 clips of *ER* and other medical dramas with emotionally charged patient-physician interactions were selected, showing histrionic, antisocial, borderline, and narcissistic personality disorders. Second part: Buckman's six-step protocol for breaking bad news to patients with recent significant loss. Students applied six-step model to 6 clips of medical dramas demonstrating strong emotional portrayal of loss, intensity, and human tragedy. 3rd year medical students (n=75 enrolled) completed pre- and post-seminar surveys (n=72 questionnaire completed) to assess knowledge and attitudes. | Research  3rd year medical students in psychiatry clerkship | 7-item pre/post seminar questionnaire (4 knowledge; 3 attitude questions). Assessed knowledge of countertransference, setting boundaries, and how to break bad news to a patient. Assessed attitude towards psychotherapy, self-reported plans for breaking bad news and what to do when student feels they have strong countertransference. | Student knowledge of countertransference, patient boundary importance, establishment of patient boundaries, and how to break bad news significantly improved from pre-seminar to post-seminar. Female students showed a significant improvement in their knowledge of countertransference, the three things to do establish patient boundaries, and the six steps involved when breaking bad news. However, no significant pre- and post- seminar difference was found in female students’ knowledge of the importance of setting boundaries or in male students’ knowledge of what to do when establishing boundaries with difficult patients. In the full group, an overwhelming majority reported improved attitudes toward a plan to break bad news and what to do when they experience strong countertransference. No significant difference between the students' reported attitudes toward psychotherapy skills as “important to all medical students to learn, no matter what area of medicine they eventually decide to pursue." Authors conclude that familiar medical dramas provided students the potential for ongoing learning and reinforcement of their learning. | 2 |
| Midmer D. Cine-ed: Using films to teach medical learners. BMJ Careers. 2004. http://careers.bmj.com/careers/advice/view-article.html?id=457 | Author presents how to use movies and television clips in teaching by identifying teaching goals, viewing conditions, and debriefing/processing. Copyright issues, precautions, and themes found in movies and television is also provided. | N/A (not a study) | Perspective  Guide for educators, but suitable for learners | N/A | N/A | N/A |
| Miller FC. Using the movie Ordinary People to teach psychodynamic psychotherapy with adolescents. Acad. Psychiatry. 1999;23:174–9. | Detailed guide highlighting the educational value of the movie *Ordinary People* in teaching principles and techniques of psychodynamic psychotherapy with adolescents, including: 1) transference and resistance, 2) neutrality and the real object, 3) slips of the tongue and observing ego, 4) unconscious conflict expressed somatically and making it conscious, 5) the role of education, 6) open- and close-ended interpretations and gratifying/frustrating patients, and 7) multiple determination of symptoms and working-through process. | N/A (not a study) | Perspective  Guide for educators, but suitable for learners | N//A | N/A | N/A |
| Misch DA. Psychosocial formulation training using commercial films. Acad. Psychiatry. 2000;24:99–104. | To describe the use movies as a tool to teach about psychosocial aspects of case formulation. | Description of psychosocial formulation training for psychiatry residents. Every 1-2 months, didactic presentation of materials and patient cases are followed by movies. Residents can elect to watch movies on their own, but most prefer to watch in the group. Facilitated discussion helps group formulate the significant biological factors and psychopathology of the case, with generated ideas written on a blackboard. Session concludes with completed formulation, emphasizing coherent, cohesive, comprehensive, cogent description of why a person is suffering from specific difficulties in thoughts, feelings, behaviours at a given time, in a given context. | Description  Psychiatry residents | No formal evaluation of this approach to teaching psychosocial formulation to psychiatry residents has yet been conducted by the authors. | Authors conclude that this teaching method is a satisfying and effective learning experience based on "enthusiastic endorsement" of the program by students, demonstrated by their request for additional sessions. Authors state that movies faithfully represent the kinds of data available to psychiatrists in their daily work, with selectivity of information, incomplete data, and information presented from a particular perspective. Movies allow residents to see in situ, not only hear about condensed, clinical case presentations. The use of movies is an affectively-charged, invigorating, and time-efficient ways to teach psychiatry residents to recognize psychopathology in the context of everyday lives of ordinary people. | N/A |
| Mischoulon D, Beresin E V. “The Matrix”: An allegory of the psychoanalytic journey. Acad. Psychiatry. 2004;28:71–7. | Description of movie The Matrix as analogy for psychotherapy. Patients understand the analogy, suggesting that The Matrix might be a suitable tool for educating psychiatry residents about the goals and functions of psychodynamic psychotherapy. | N/A (not a study) | Perspective  Psychiatry residents | N/A | N/A | N/A |
| Muñoz-Crego Á, Rodríguez YS, Prado RS. MicroDeCine: The teaching of Clinical Microbiology at the University of Santiago de Compostela by means of film. J. Med. Movies. 2009;5:87–91. | To improve students' attitude and motivation in the study of infectious diseases by teaching the topic using movies (Moulin Rouge-tuberculosis, Out of Africa-syphilis, Panic in the Streets-plague, Trainspotting-AIDS, In Enemy Hands (UBoat)-meningitis, The Painted Veil-cholera, In Love and War-infection of wounds/soft tissue, Outbreak-bioterrorism). | Elective course "MicroDeCine" with 2 movie screenings per week (Mondays and Wednesdays), over 4 weeks. 8 movies focusing on different infectious disease topics were screened. Attendance was kept throughout with a survey completed on the last day. Average of 179 students per screening session, 114 students completed the survey. | Research  Medicne,  Biology,  Pharmacy,  Odontology,  and Nursing students | Survey assessed student satisfaction with the course and knowledge about infectious diseases. | Students were very satisfied with MicroDeCine (15% Good, 40% Very Good, 45% Excellent) and most students reported that the course increased their interest in clinical microbiology (89% yes, 11% no). The majority of students felt they learned something new about infectious diseases (92% yes, 8% no). Data was also collected on students' favourite topics (cholera and AIDS rated highest), suggested topics for future sessions, and logistical questions about best month for the course and timeframe (64% favoured the 2/week over 4week screening method). Authors state that teachers must transmit information through attractive methods and movies are a great tool that should not be overlooked. This study surveyed student self-reports of knowledge increase; future studies could deliver pre/post tests for objective assessment of knowledge change in infectious disease topics. | 1, 2 |
| Murphy-Shigematsu S, Grainger-Monsen M. The impact of film in teaching cultural medicine. Fam. Med. 2010;42:170–2. | To teach students about cultural sensitivity and improve health care for culturally diverse patients through the use of a documentary *(Hold Your Breath*). | The film is screened in its entirety, followed by question-and-answer with the producer. Participants are then divided into small groups, with a trained and knowledgeable facilitator, for roundtable discussions about the film and related issues of health care of immigrants and minorities. | Description  Medical students | No description of outcome directly measured. Authors make a general statement about written evaluations. | Authors conclude that students enjoyed the format and summarized some of their responses (but these responses are never tabulated or provided in full). Students report increased cultural and religious awareness and find the use of documentary medium efficient in revealing complex situations and perspectives in a short amount of time, and is emotionally engaging. Authors state other people who have reported the use of the documentary have "favorable comments as to its impact." | 1, 2 |
| Ozcakir A, Bilgel N. Educating medical students about the personal meaning of terminal illness using the film, “Wit.” J. Palliat. Med. 2014;17:913–7. | To test the relevance and usefulness of the movie *Wit* in teaching medical students about the personal meaning of terminal illness and to assess the impact of this teaching method on students’ attitudes toward palliative care. | First year medical students (n=518) were divided into 8 subgroups of 25-30 students. They shown the entire movie *Wit,* which deals with a terminal illness and death. Post-film viewing, they filled out an evaluation questionnaire, answered questions about the film (close-ended) and shared their thoughts/feelings about palliative care (open-ended questions). | Research  1st year medical students | Close-ended questions about the film assessed student rating of the film, emotional impact, type of emotion (positive/neutral/negative), and usefulness of the film. Open-ended questions solicited descriptions of which scenes affected students most emotionally, the effect of the film on personal interest in caring for dying patients/learning more about palliative care, and what features they would improve in palliative care scenario depicted in the movie. | 88% rated the film as excellent, very good, or good. 54% said emotions of terminally ill patients were fully portrayed in the film and in a very realistic way. Approximately 61.4% of the students found this film emotional. Most students (80.5%) stated that this film made them think about the emotional and spiritual suffering that dying patients go through and found this learning approach about palliative care more useful than didactic lectures and journal article readings, but not more useful than bedside rounds. 65.3% thought that caring for dying patients would be very or fairly personally satisfying. No questions about their knowledge pre/post session were assessed, but subjective student comments that they learned more about palliative care. Authors conclude that movies can be used as an innovative, effective method to provide training about end-of-life issues and doctor–patient communication even in the earlier stages of medical education. | 1, 2 |
| Pais de Lacerda A. Medical education: Addiction and the cinema (drugs and gambling as a search for happiness). J Med Mov. 2005;1:95–102. | To evaluate the use of movies as a teaching tool to help students develop a better understanding of addiction (substance use and gambling). | Authors provide details and themes for 5 movies related to different addiction topics (*Trainspotting, Requiem for a dream, Leaving Las Vegas, Kids, and Rounder*s). Description of extracurricular, 1-day course on addictions where movie clips were screened, followed by discussion which attended to treatment for scenarios presented, while encouraging considerations of individual, social, and medical aspects of addictions represented. 5-point scale multiple choice questionnaire with open-ended questions were completed by all student participants (n=35). | Research  4th and 5th year medical students | Student satisfaction and level of interest in the course. Student knowledge and attitudes towards use of movies in teaching/learning about addictions. | The majority of the students reported that the movie clips were adequately chosen and related well to the addiction themes, stimulated discussion, cultivated their interest, addressed themes relevant for their professional activities, and changed their attitude about drug addiction. | 1, 2 |
| Pavlov A, Dahlquist G. Teaching communication and professionalism using a popular medical drama. Fam. Med. 2010;42:25–7. | To teach communication and professionalism using television medical dramas. | Brief description of *Grey's Anatomy* episode selected as an example of medical drama depicting communication and professional challenges. Specific clips are described with questions to: 1) explore reactions to patients “late to treatment,” 2) practice and gain more comfort in responding to questions about life-threatening situations, 3) discuss ways to manage personal feelings in response to difficult situations, and 4) manage appropriate informed consent with vocal family members. Residents (n=9) completed a post-presentation evaluation. | Description  Family practice residents | Student reactions to the use of medical drama clips and discussion to learn about communication and professionalism. | Not a detailed description of assessment, but surveys were completed immediately after the conclusion of the presentation, with all residents rating the use of medical clips stimulating interest and discussion as "4" or "5" (with "5" being "extremely helpful). Residents would like to see clips used in the future and said the use of clips added to their appreciation of the topics. | 1 |
| Quadrelli S, Colt HG, Semeniuk G. Appreciation of the aesthetic: A new dimension for a medicine and movies program. Fam. Med. 2009;41:316–8. | To illustrate specific clinical and epidemiological aspects of medicine but also to promote discussions of professionalism, compassion, medical ethics, and social injustice through movies. | Brief description of monthly Medicine and Movies program. Comprised of suggested readings, film or movie vignette viewing, and guided group discussions. | Description  Senior medical students | Major purpose was to help students critically appraise the clinical relevance of a film’s story, but also appreciate technical and aesthetic values of each movie. | No clear description of how outcome/evaluation was measured. Authors state that movies present patient narratives, which heightens students' understanding of situational ethics and human suffering. Appreciation of aesthetic value of film increases thoughtful awareness of movies' messages and reinforces humanistic perspectives. | N/A |
| Raballo A, Larøi F, Bell V. Humanizing the clinical gaze: Movies and the empathic understanding of psychosis. Fam. Med. 2009;41:387–8. | A brief editorial arguing that movies can be effective in demonstrating psychological disturbances/presentations to medical trainees as a sophisticated alternative to biographical narratives, facilitating the development of empathic skills. | N/A (not a study) | Perspective  Editorial of interest to educators and learners. | N/A | N/A | N/A |
| Rabow MW, Goodman S, Chang S, Berger M, Folkman S. Filming the family: a documentary film to educate clinicians about family caregivers of patients with brain tumors. J. Cancer Educ. 2010;25:242–6. | To evaluate the educational value of a documentary film about family caregiving for patients with brain tumors (*The Caregivers*). | Pre–post survey among neurosurgeons, neuro-oncologist, and other clinician viewers. Survey assessed film quality and attitudes toward family caregivers as well as any change in these attitudes after viewing the film. Elective screenings for UCSF students enrolled in class about cancer and screening at national conferences for neurosurgeons, neuro-oncologists, interdisciplinary care teams, and medical educators. | Research  Neurosurgery/neuro-oncology clinicians and medical students. | Pre-post attitudes about family caregivers' impact on health of patients and evaluation of film's quality. | Viewers came away with stronger beliefs that “all families of patients with brain cancers should meet with a social worker” and that “family caregivers greatly impact the health of patients.” Viewers were more likely to disagree that “supporting family caregivers is primarily someone. All viewers felt that the film was of high quality (9.27 on a ten-point Likert scale) and important (9.03), that they learned something useful from the film (8.67), and that the film is an effective way to teach about family caregivers (8.98), and should be seen by all clinicians caring for patients with brain tumors (9.23). | 1, 2 |
| Ramchandani D. The downside of teaching psychopathology with film. Acad. Psychiatry. 2012;36:154–5. | Author posits that movies should not be used to teach psychopathology because of stereotypical or oversimplified representations. Using movies to illustrate psychopathology may mischaracterize mental illness and inadvertently lead to greater stigmatization of psychiatry as a profession. Author provides negative examples of psychopathology in movies to support his cautionary point. | N/A (not a study) | Perspective  Commentary for educators, but may be of interest for learners. | N/A | N/A | N/A |
| Retamero C, Walsh L, Otero-Perez G. Use of the film The Bridge to augment the suicide curriculum in undergraduate medical education. Acad. Psychiatry. 2014;38:605–10. | To evaluate the use and reception of the movie *The Bridge* as complementary to the suicide curriculum of medical students. | Second year medical students (n=196 enrolled) watched clips focusing on suicide from the movie *The Bridge* as part of neuroscience curriculum in understanding suicide. Lecture and discussion accompanied movie clip viewing. Pre/post intervention questionnaire on attitudes about suicide and use of movies as an educational aid filled out by most of the students (n=180). | Research  2nd year medical students | Student attitudes towards suicide and satisfaction about movie as an educational tool. | Students believe movies have value in helping them understand suicide (pre 90.6%, post 89.4%). Agreed that watching and discussing a film reinforced concepts learned in the lecture (pre 92.2%, post 88.3%), and disagreed that using films for education was a waste of time (pre 90.6%, post 91.1%). Unpaired questions on post-movie survey indicated after watching the movie, students felt they had a better understanding of the risk factors for suicide (85% agreed), understood the stigma faced by survivors of suicide (85.0% agreed), and understood what the last days of a suicidal patient were like (90.0% agreed). *The Bridge* represents a useful method for teaching students about suicide and provides an additional means for helping them gain a deeper understanding of suicide. | 1, 2 |
| Rieder RO. Teaching about schizophrenia. Schizophr. Bull. 1974;5–9. | To review what is known and what has been said about schizophrenia from many different viewpoints. Topics will be chosen from the following: symptomatology, subjective experience, diagnosis, genetic research, biochemical research, family interaction research, pharmacological therapy, and psychotherapy. | 25 people registered for a course on schizophrenia, broken down into 4 topics: phenomenology, subjective experience, research, and therapy. Course based on readings, movies (*Breakdown*), discussion, and final exam. | Description  Open course at NIH, researchers, nurses, graduate students, others joined (including housewives and drug salesman) | Final exam tested student knowledge through long answer questions on generalizations about schizophrenia, with students having to provide written examples discussing phenomenology, terminology, and research on schizophrenia. | No outcome/evaluation assessed. This article focuses on description of author's course syllabus. The final exam questions are included, but the author does not explain exam results and whether/how students reacted or whether/how student attitudes or knowledge changed. | N/A |
| Rosenstock J. Beyond a beautiful mind: Film choices for teaching schizophrenia. Acad. Psychiatry. 2003;27:117–22. | To describe benefits of using commercially available films to teach about schizophrenia, using *A Beautiful Mind* and *Clean, Shaven* as examples. Includes a table of 20 films with summary/comments for teaching about schizophrenia. | N/A (not a study) | Perspective  Guide for educators, but suitable for learners | N/A | N/A | N/A |
| Ross PT, Kumagai AK, Joiner TA, Lypson ML. Using film in multicultural and social justice faculty development: Scenes from Crash. J. Contin. Educ. Health Prof. 2011;31:188–95. | To evaluate the impact of an interactive faculty development workshop on faculty awareness, skills, and confidence to facilitate, manage, and stimulate discussions about controversial issues related to the multicultural classroom, which will in turn prepare medical students to address health care disparities based on visible and invisible social determinants (eg. race, ethnicity, gender, religion, class, geographical origin). | Workshop using train-the-trainer model, designed for interactivity and engage participants (n=25) in critical reflection through viewing scenes from movie *Crash,* open discussion, reflective writing, small-group/pair sharing, and fishbowl technique (women discussed responses in a circle while men listened). | Research  Faculty members | Survey evaluations completed post-workshop (n=18) with open- and close-ended questions. Faculty feelings regarding benefits of discussions of race and diversity and how they felt about conflict in the classroom setting and various strategies to maintain effective classroom dialogue in spite of conflict. Reflective writing exercises also used as evaluation data. | Participants reported mixed feelings regarding the use of conflict in the classroom to stimulate discussions around race, with 44% neutral and 61% indicating reluctance to discuss race/class because of possible conflicts. Despite feelings of discomfort, 72% indicated it is more important for small-group members to challenge each other's beliefs rather than for group members to get along. 78% responded that the workshop would help them better understand the perspectives of their students, and 89% indicated that being confronted with unfamiliar perspectives and situations helped them to reflect on issues of race and diversity in US society. Participants reported the movie caused them to think about issues in new ways and reconsider their own views and attitudes toward others. | 1, 2 |
| Self DJ, Baldwin DC. Teaching medical humanities through film discussions. J. Med. Humanit. 1990;11:23–37. | To discuss the importance and appropriateness of film to promote the affective focus of medicine on the relief of suffering and description of an elective course to teach an ethical ideal of caring through weekly film discussions. | An elective course (pass/fail) conducted as an informal, noontime, brown bag lunch session. There were 18 participants. No didactic presentations or lectures. Each week, a short film was screened followed by group discussion for remainder of the hour. Discussions led by medical humanities faculty member with philosophical background. Topics covered included prejudice and freedom of speech to methods of advertising and sexuality of the elderly, with emphasis on issues of reducing prejudice and increasing tolerance of others' differing values. | Description  1st and 2nd year medical students | No description of outcome directly measured. Authors make an evaluative statement about student satisfaction with the course, but no data presented. | No clear description of how outcome/evaluation was measured. Authors state that based on the quality of discussion and "judgment of the students and the instructor," the course was "enormously successful." Authors posit that the film discussion elective is an affective activity emphasizing the development of ethical ideal of caring, tolerance, and reduction of prejudice (compared to usual teaching of medical ethics, which is primarily cognitive emphasizing development of a code of principles such as justice, autonomy, and beneficence). Authors state the closeness between students and faculty role-modeled the course objectives of creating an ethical ideal of caring, relatedness, sensitivity to others, and that students engaged in significant introspection, reflection, critical self-analysis of values. | N/A |
| Self D, Baldwin D, Olivarez M. Teaching medical ethics to first-year students by using film discussion to develop their moral reasoning. Acad. Med. 1993;68:383–5. | To evaluate teaching medical ethics using film discussions to develop students' moral reasoning. | Pretest/posttest design with control group. Elective course on social issues in medicine with weekly 1 hour discussion of short film: Fall quarter participants (n=48), Fall & Winter quarter participants (n=37), and control group who did not take the course and had no exposure to film discussions (n=29). | Research  1^st^ year med students (n=114) | Analysis of moral reasoning skills using Defining Issues Test (DIT). | Both groups of registrants showed increased moral reasoning posttest scores with intervention (film discussion elective) compared to control group. Must keep in mind self-selection bias as students with higher levels of moral reasoning appear more likely to elect additional courses to further develop moral reasoning skills. Film discussion is an appropriate method for teaching medical humanities with increased moral reasoning skills as one of the major objectives. | 1, 2 |
| Shevell AH, Thomas A, Fuks A. Teaching professionalism to first year medical students using video clips. Med. Teach. 2014;1–8. | To examine medical students’ perceptions of using video clips as a beneficial teaching tool to learn professionalism and other aspects of physicianship. | Mandatory Physician Apprenticeship course of 30 Osler groups and 175 1st year students. Delivered multimedia teaching modules containing selected brief (less than 5 minute) video clips from the television medical drama *ER,* accompanied by written teaching guides. Group discussion with Osler fellow and co-leaders. Questionnaire with 5 open-ended questions completed immediately following viewing of the video clips and group discussion. Responses were interpreted using qualitative description and thematic analysis to evaluate the educational merits of this format. | Research  1st year medical students | 5 open-ended questions to assess student reaction to scenarios and students' perceptions of effectiveness of video clips to encourage reflection and discussion. | 21 (out of 30) Osler groups submitted evaluation forms, total of 112/175 students (64% response rate). Themes garnered from student comments regarding utility of video clips included: authenticity and believability, thought provoking, skills and approaches, setting, medium, level of training, mentorship, experiential learning, effectiveness, and relevance to practice. Themes from student responses to attributes of physicianship portrayed in video clips included: patient-centeredness, communication, physician-patient relationship, professionalism, ethical behaviour, interprofessional practice, and mentorship. This study elucidates student reactions to television clips in teaching physicianship qualities. Students perceived value in this methods as a means of learning professionalism and other aspects of physicianship. | 1 |
| Sierles FS. Using film as the basis of an American culture course for first-year psychiatry residents. Acad. Psychiatry. 2005;29:100–4. | To teach international medical graduate (IMG) psychiatry residents about aspects of American culture: 1) African American culture, 2) military culture, 3) gender, 4) Jewish American culture, and 5) socioeconomic status. | 3 hour course delivered yearly from 1995-2004 (8 years; 2 years no data). Course format: 2hrs for movie screening + 30-45 post-movie discussion. Author describes the movies chosen and how they relate to teaching each of the 5 themes. Pre/post-course multiple choice exam and anonymous course ratings (outstanding, strong, competent, failing). 3 department administrative staff participated anonymously, as hypothesized they would score higher than PGY-1 IMGs since the department staff have lived in the United States their whole lives. | Research  IMG psychiatry residents  Department administrative staff | Student attitudes towards the course and student knowledge of 5 aspects of American culture. | High student satisfaction with course and use of movie clips (over 8 years, 76.7% rated outstanding; 19.1% strong; 2.1% competent; 2.1% failing). Only one student in one year (2003) gave a low rating and approached instructor voluntarily to say that they found the course mistakenly stereotyped African Americans as having anti-white attitudes. No other residents in any year wrote or said that they felt the course was biased. Pre-course test mean score significantly higher for staff members (72.2%) than mean score of IMG residents (49.9%). Post-course mean score for IMG residents plus 1 US medical graduate resident was 80.3%. The author concludes that movies can form the basis of a valuable acculturation course. | 1 , 2 |
| Sondheimer A. The life stories of children and adolescents: Using commercial films as teaching aids. Acad. Psychiatry. 2000;24:214–27. | Authors provide a guide to a list of movies that can be used to teach about topics in child and adolescent psychiatry. A request was sent out to members of the Association for Academic Psychiatry asking for ideas on what movies would be beneficial from an educational standpoint on facets of child/adolescent development, pathology, and/or responses to the environment. Over a 2-year period, the list collected was analyzed by the authors to select a large number of movies that might be useful. Description of movies and themes is presented, a long appendix listing the movies presented, which authors hope will serve as a useful teaching tool and reminder of the value of using teaching aids and approaches that are "out of the box." | N/A (not a study) | Perspective  Detailed catalogue of movies aimed at educators, but suitable for learners | N/A | N/A | N/A |
| Spike J. Television viewing and ethical reasoning: why watching Scrubs does a better job than most bioethics classes. Am. J. Bioeth. 2008;8:11–3. | Author suggests using *Scrubs* to teach ethical issues with viewing of medical drama episode followed by discussion. In/accuracies in the show are grist for good discussion. Clips may be useful, but it is useful for audience to see the whole story with all motivations and lessons, which may be more helpful for making learning stick. Provides examples in *Scrubs* that can be used effectively to teach ethics to medical students. | N/A (not a study) | Perspective  Commentary and suggested uses of *Scrubs* for medical educators. | N/A | N/A | N/A |
| Tarsitani L, Brugnoli R, Pancheri P. Cinematic clinical psychiatric cases in graduate medical education. Med. Educ. 2004;38:1187. | Cinema frequently describes characters with mental health disorders, providing, in high profile movies, realistic, subtle and attractive case histories. Popular movies can fill the need for high quality clinical cases to develop differential diagnosis & treatment skills in area of general psychiatric practice. | Five 2 hour interactive sessions alternated with frontal lectures. Each segment was followed by multiple-choice questions about differential diagnosis, prognostic evaluations, treatment options and other clinical issues. A show of hands to the questions was followed by interactive group discussions among residents, with facilitation by teachers. PowerPoint presentations were used to show diagnostic criteria, guidelines and treatment algorithms, as well as outlines of scientific evidence regarding every question, in order to verify opinions and to provide scientific information in response to doubts. | Research  Year 2 Psychiatry residents | At the fifth clinical case (session), residents (n = 10) were asked to rate on a 5-point Likert scale (1 = strongly disagree, 5 = strongly agree), the usefulness and relevance of the sessions and their evaluation of them. | All respondents agreed or strongly agreed with the statement: Cinematic clinical cases are useful and should be a required part of the clinical psychiatry course (mean = 4.8, SD ¼ 0.4). Resident feedback consistently included an appreciation of projected movies and a willingness to participate in further sessions in the Year 3 course. | 1 |
| Tobia A, Draschil T, Sportelli D, Katsamanis M, Rosenberg S, Williams JM. The horror!: A creative framework for teaching psychopathology via metaphorical analyses of horror films. Acad. Psychiatry. 2013;37:131–6. | To describe REDRUM (Reviewing [Mental] Disease with a Rudimentary Understanding of the Macabre), a course to enhance learning through creative discussion of examples of psychopathology that come from film and literature. | 15 week course - 1 hour didactic instruction supplemented with movie discussion or excerpts from literature. The psychopathology examples from film and literature were discussed in a metaphorical context to prevent perpetuation of mental illness/patients as "monsters." | Description  Psychiatry residents | No description of outcome measured or reported. | Authors give detailed description of modules and movies chosen. Authors allude to anonymous course evaluations that residents are encouraged to complete to address feelings of being offended, but no clarification about reactions to course format/content or knowledge change in residents' understanding of psychopathology. | N/A |
|  |  |  |  |  |  |  |
| Trachtman H. The medium is not the message. Am. J. Bioeth. 2008;8:9–11. | Argues against using television medical dramas as a tool for teaching ethics to medical students. One should question the staying power of lessons learned from medical dramas. There are intrinsic flaws in the way these dramas present bioethical problems that makes them suspect material to teach medical and nursing students how to think about and resolve bioethical problems in clinical practice. Problems are sensationalized, details about problems arising out of heterogeneous population/social context may be ignored, and dramatic problems solved in an hour overlook the hard work involved in handling of routine bioethical problems. | N/A (not a study) | Perspective  Commentary for medical and health professional educators. | N/A | N/A | N/A |
| Tsai MH. Movie dialogues as discourse data in the study of forecasting mechanisms in the delivery of medical bad news. Commun. Med. 2013;10:165–75. | To apply Schegloff's (1988) and Maynard's (2003) frameworks of forecasting mechanisms to analyze bad news delivery depicted in movie dialogues. Describes 3 movies that can be used to teach students how to break bad news (*Saving Private Ryan, Living Proof, Fatal Attraction*). Author does not detail whether the suggested intervention was ever delivered. | N/A (not a study) | Perspective  Suitable for educators & learners | N/A | N/A | N/A |
| Volandes A. Medical ethics on film: Towards a reconstruction of the teaching of healthcare professionals. J. Med. Ethics. 2007;33:678–80. | Volandes suggests how documentary film can be used to teach medical ethics. In Volandes' opinion, film can improve the present method of using clinical vignettes. | N/A (not a study) | Perspective  Suitable for educators & learners | N/A | N/A | N/A |
| Wadhwa R, Thakur JD, Cardenas R, Wright J, Nanda A. Synoptic philosophy in a neurosurgical residency: A book and cinema club. World Neurosurg. 2013;80:e21–5. | To combine neurosurgical training with humanities education through a Book and Cinema Club. While humanities is not one of the six core ACGME competencies, a wider worldview and better understanding of human spiritual values and emotions provided through humanities education clearly support interpersonal and communication ACGME competencies. | Book Club held every few months, followed a journal club format (relaxed atmosphere that allows for exchange of ideas). Residents summarize and express individual opinions about the selected book or movie, followed by nonhierarchical group discussion. Post-intervention subjective questionnaire with yes/no questions given to residents who participated (n=13) | Research  Neurosurgery residents | Student satisfaction with Book and Cinema Club topics, whether discussions have helped in neurosurgery residency, and whether it should be included as part of curriculum. | 61.5% believed that the topics discussed in the Book and Cinema Club were pertinent to their everyday life. 77% of the residents believed that the Book and Cinema Club had helped them in the neurosurgical residency in some way. However, most residents (7 of 13) did not think that Book and Cinema Club should be included as a part of the curriculum in the 80-hour work week, although 4 of the 7 thought that it should be included as a supplement to the 80-hour work week. Future study with prospective design needed to verify these findings. | 1 |
| Walter G, McDonald A, Rey J, Rosen A. Medical Student Knowledge and Attitudes Regarding ECT Prior to and After Viewing ECT Scenes from Movies. J. ECT. 2002;18:43–6. | To assess medical students’ level of knowledge and attitudes regarding ECT at the commencement of a psychiatry term and to examine the effects of brief depictions of ECT in movies on the students’ knowledge and attitudes. | Used five 30-90 second movie clips to demonstrate the portrayal of ECT and evaluated students’ views on the procedure. 26-item questionnaire about demographics, knowledge and attitudes towards ECT, impressions of movie portrayal of psychiatry generally and ECT in particular, and which movies (from a given list) students had already seen. Students also asked if they approved certain treatments: ECT, psychiatric medication, "talking therapies," and psychosurgery. Questions on ECT indications, side effects, technique, and stigma, were then interspersed with the movie clips. One question was posed before and after the clips: what advice the student would give a family member or friend who was offered ECT. | Research  4th year medical students (sample in London, UK, n=26)  2nd year medical students (sample in Australia, n=68) | Student knowledge and attitudes towards ECT and movie portrayals, indications for ECT, treatment procedure, and side effects. | Sources of knowledge about ECT indicated by students were reading (51%), followed by film/television (46%). 23% of students thought ECT was safe, while 62% who did not know. Less than one third (30%) believed ECT to be effective, while 55% did not know. The majority of students thought ECT had poor public image (84%) and that the portrayal of psychiatry in movies was inaccurate (70%). The majority of students (85%) agreed or strongly agreed that psychiatrists should attempt to influence positively the portrayal of psychiatric treatments in movies and television. Levels of knowledge about the indications, side effects, and mode of administration were poor, and attitudes were gene ally negative. Viewing the ECT scenes influenced attitudes toward the treatment; after viewing, one-third of the students decreased their support for ECT, and the proportion of students who would dissuade a family member or friend from having ECT rose from less than 10% to almost 25%. | 2 |
| Weber CM, Silk H. Movies and medicine: An elective using film to reflect on the patient, family, and illness. Fam. Med. 2007;39:317–9. | To aid students in gaining gain an appreciation of the many issues that face a patient and his/her family when an illness is present. 5 themes with specific movie choices for each are described: (1) the family & illness, (2) the family & loss, (3) the family & caregiving, (4) the family & substance abuse, and (5) extended family and illness. | Elective, small group sessions with film viewing, discussions about medical issues related to how illness affects the family, weekly journal reflections, and 15-minute presentations by students with essay to summarize experience. | Description  Medical students | Through an anonymous survey, students provided subjective evaluation of their enjoyment of the use of movies to teach about family and illness. | No detailed outcome/evaluative data. Authors provide selected quotes from students who state they are more knowledgeable about diverse ways illnesses' impact on patients/families and that they enjoyed the use of film. Authors state that film offers more insight for students into patient's life/families and encounter complex family scenarios they may not encounter for many years in real life. Authors note watching entire movies (not only clips) allowed students to connect with characters more as complex people rather than teaching props. | 1, 2 |
| Welsh CJ. OD’s and DT's: Using movies to teach intoxication and withdrawal syndromes to medical students. Acad. Psychiatry. 2003;27:182–6. | To evaluate students' perceived benefit of the inclusion of movie clips in learning about intoxication and withdrawal syndromes. | Commercially available movie clips were edited together and shown as part of a 100 minute lecture on intoxication and withdrawal symptoms. 122 attended with 89 medical students filling out anonymous questionnaire (response rate 73%). Post-lecture questionnaire only. | Research  2nd year medical students | Student attitude towards how movie clips would help them remember various intoxication and withdrawal symptoms. Student knowledge about and ability to recognize intoxication and withdrawal from various substances, and knowledge about medical severity. | Almost all students felt that the clips would help them remember various intoxication and withdrawal symptoms (92% rated 4 or 5; none rated 1 or 2). Student rated themselves less knowledgeable about intoxication and withdrawal from various substances prior to session (21% rated 4 or 5; 33% rated 1 or 2). A significant rated increased knowledge post-session in comfort recognizing symptoms (88% rated 4 or 5; none rated 1 or 2) and better able to appreciate the medical severity of some of the intoxication and withdrawal symptoms (93% rated 4 or 5). Qualitative comments at end of questionnaire included that movie clips "will stick in my mind" and "kept me interested." Author concludes that societal stereotypes and personal experiences tend to influence student's attitudes and, consequently, openness to learning about addiction. The use of popular film can help to make this process more interesting and help to reduce some of the negative bias while at the same time conveying factual information. | 1 , 2 |
| White GB. Capturing the ethics education value of television medical dramas. Am. J. Bioeth. AJOB. 2008;8:13–4. | Author discusses the appeal of medical dramas and that given television shows are relevant to the experiences of medical and nursing students, then there is the potential to use medical dramas for ethics education. Author suggests ways to facilitate this include asking students to watch a show and identify ethical issues or describe ethics decision-making processes employed. A longer assignment might be an essay on the "good nurse/physician" as exemplified (or not) in medical dramas. Study guide and teaching strategies can be developed to capture the opportunity that fictional medical dramas with real life features to be used for educational purposes. | N/A (not a study) | Perspective  Commentary for medical and health professional educators. | N/A | N/A | N/A |
| Wicclair MR. The pedagogical value of House, M.D.--can a fictional unethical physician be used to teach ethics? Am. J. Bioeth. AJOB. 2008;8:16–7. | Author discusses the problematic aspects of a medical drama like *House*, with specific examples. Identifies the potential uses of House's unethical behaviour to educate medical students in ethics, including effective communication with patients, the value of patient autonomy, the basis for ascertaining a patient's best interests, and limitations of paternalism. As long as medical students can discern reality from fiction, there may be no need for concern about the show's negative influence, and *House* can in fact be used as audio-visual teaching aids for ethics education. | N/A (not a study) | Perspective  Commentary and suggested uses of *House* for medical educators. | N/A | N/A | N/A |
| Wilson N, Heath D, Heath T, Gallagher P, Huthwaite M. Madness at the movies: Prioritised movies for self-directed learning by medical students. Australas. Psychiatry. 2014;22:450–3. | To assemble a list of 10 movies to facilitate self-directed learning in psychiatry by medical students. | Authors identified top 4 mental health conditions from Global Burden of Disease 2010 study: depressive and anxiety disorders (amalgamated as one category), illicit drug use disorders, alcohol use disorders, and schizophrenia. Systematic identification of prospective movies followed (503) identified. Based on highest artistic and entertainment value (using Rotten Tomatoes, movies with over 75% rating) and movies from 1960 onwards, 23 movies were selected for further viewing and ranking. Final selection of 10 movies with at least 2 movies per topic area. | Research (review)  Guide for educators, but suitable for learners | N/A | Authors provide a guide to 10 systematically chosen movies covering the 4 psychiatric/substance use topic areas. Authors state that these movies can be used for independent learning purposes in psychiatry. Authors suggest further research to evaluate self-directed learning of medical students by viewing the movies. | N/A |
| Winter RO, Birnberg BA. Family systems at the movies. Fam. Med. 2005;37:96–8. | To describe the use of movies in teaching family medicine residents about different family dynamics. | Description of sessions which combine reading with movie clips and discussion to heighten residents' understanding of multi-generational issues, importance of genograms, impact of family secrets and family dynamics, effect of stress on family life, and the attributes of a functional family. Movies and themes addressed are described (*Joy Luck Club, Shine, Secrets and Lies*). | Description  Family medicine residents | No formal evaluation or assessment conducted. | Authors did not report any evaluation of student satisfaction, but state that the teaching method was well received based on empathic reactions to characters during discussion. Authors believe experiencing and analyzing "film families" result in residents better prepared to understand and take care of actual patient and families, though no formal measure of knowledge was reported. | N/A |
| Wong RY, Saber SS, Ma I, Roberts JM. Using television shows to teach communication skills in internal medicine residency. BMC Med. Educ. 2009;9:9. | To assess the efficacy of cinemeducation (using medical dramas) for teaching communication skills using the Kalamazoo model, which has not been used in previous studies. | 1 hour interactive session on communication with brief didactic presentation on communication competencies, 3 excerpts from *House* and *Grey's Anatomy* featuring 3 clinical scenarios related to communication (addressing end-of life issues, attending to psychosocial aspects of illness, and disclosing medical errors), and 7 questions that mirrored the 7 elements in the Kalamazoo's model. interactive reflection period followed where residents were encouraged to input their ideas on the excerpts verbally and discuss what they would have done differently in each of the featured scenarios. Pre- and post-test were administered. Responses based on 5-point Likert scale. 64 residents attended and 43 (67%) responded. | Research  Internal medicine residents | Student satisfaction at the completion of the session and knowledge of the Kalamazoo model competencies. | At pre-test, only 23% of respondents indicated that they had a good, very good or excellent understanding of an evidence-based communication model such as the Kalamazoo, whereas this proportion improved to 93% in the post test (P < 0.0001). The residents' self-reported comfort in applying an evidence-based communication model to actual clinical encounters improved substantially, from 23% who indicated good, very good or excellent level in the pre test to 88% post test (P < 0.0001). Resident satisfaction at the completion of the session was encouraging as 80% indicated that they would like to participate in this form of communication teaching in the future. | 1, 2 |
| Yamada S, Maskarinec G, Greene G. Cross-cultural ethics and the moral development of physicians: Lessons from Kurosawa’s Ikiru. Fam. Med. 2003;35:167–9. | To raise students' awareness of the cultural nature of ethical principles and exercise their moral reasoning. | Brief description of Kurosawa's *Ikiru* used at two points in medical education: a movie clip is shown to 1st year students during PBL colon cancer tutorial; whole movie is discussed during 3rd year Family Medicine clerkship | Description  1st year medical students  3rd year Family Medicine clerkship | Student perception about moral and ethical questions by examining problems confronting protagonist Watanabe. Student recognition that their values might be different from those of others from different cultures. | No outcome/assessment data reported, so no evaluation apparent.. Authors quote some of the students' ethical and existential questions after viewing the movie. Authors suggest the movie helps students understand moral trajectory of illness and their own responsibilities as moral actors providing healing in all dimensions. | N/A |
| Zerby SA. Using the science fiction film invaders from mars in a child psychiatry seminar. Acad. Psychiatry. 2005;29:316–21. | To describe the use of a science fiction movie (*Invaders from Mars)* to teach principles of child development, clinical features of separation anxiety and nightmares, and clinical interventions including child psychotherapy, child protective issues, and crisis management. | Lengthy description of synopsis and analysis of the film addressing specified child psychiatry topics. Author mentions the use of the movie and discussion points in film seminar. No data presented for specific number of participants. | Description  Psychiatry residents | No formal evaluation or assessment conducted. Author uses sample student quotes, but no description of outcome/evaluation measures. | No clear description of how outcome/evaluation assessed, though author gives sample comments from residents, all of which are positive ("learned much about development," provoked "a great deal of thought [about] the psychodynamic piece," and "great medium to teach"). Author concludes that psychiatry residents have found the use of *Invaders* and other movies to teach aspects of child psychiatry to be useful and memorable. | 1, 2 |
